# Supplementary material for: A molecular extraction process for vanadium based on tandem selective complexation and precipitation
Source: Nat Commun. 2024 Mar 23;15:2614. doi: 10.1038/s41467-024-46958-6 (PMC10960790; doi:10.1038/s41467-024-46958-6)
Supplement: Supplementary file 1 — Supplementary Information [file 41467_2024_46958_MOESM1_ESM.pdf]

A molecular extraction process for vanadium based on tandem selective complexation and precipitation

Oluwatomiwa A. Osin[1], Shuo Lin[1], Benjamin S. Gelfand[1], Stephanie Ling Jie Lee[2,3], Sijie Lin[2,3], George K. H. Shimizu[1] *

[1]Department of Chemistry, University of Calgary, Calgary, Alberta T2N 1N4, Canada

[2]College of Environmental Science and Engineering, Biomedical Multidisciplinary Innovation Research Institute, Shanghai East Hospital, Tongji University, 1239 Siping Road, Shanghai 200092, China

[3]Key Laboratory of Yangtze River Water Environment, Shanghai Institute of Pollution Control and Ecological Security, Tongji University, 1239 Siping Road, Shanghai 200092, China

*Corresponding author. Email: gshimizu@ucalgary.ca

Supplementary Discussion 1. Synthesis and characterization of H_2CID^{III}

In consideration of practical applications, improving H_2CID^{III} synthesis aimed to increase yield, reduce synthesis duration and energy consumption. In previous reports, the synthesis pathway entailed a three-step process, commencing with acenapthenequinone sulfamation via acid-catalyzed condensation. Subsequently, a high-energy-demanding cyanation process at 180°C was used to eliminate sulfur dioxide and furnish the dinitrile intermediate. Finally, hydroxylamination of the dinitrile was conducted to yield the desired product, leading to an overall yield of 28% within a time span of approximately four days. In contrast, the improved synthesis procedure used 1,8-dibromonapthalene (dibromide) as starting material, resulting in a 2-step approach, that could be accomplished within a span of 24 hours (as depicted in Supplementary Fig. 3). Cyanation of aryl halides via the Rosenmund-von Braun Reaction[1] was adopted and performed under atmospheric conditions in dimethylformamide (DMF) at 130 °C to afford 1,8-naphthalenedicarbonitrile (dinitrile, yield = 84%). Notably, the excess copper cyanide could be recovered, enabling its reuse for subsequent syntheses, and eliminating the potential for environmental pollution and material wastage. Subsequently, the dinitrile was treated with aqueous hydroxylamine to give H_2CID^{III} in high yield (94%), and an overall yield of 79%. The yield (Y) was calculated using the formula:

$$\mathrm{Y}\,(\%) = \frac{\text{the experimentally obtained quantity of } \mathrm{H_2CID^{III}}}{\text{the maximum possible quantity of } \mathrm{H_2CID^{III}}} \times 100 \qquad (1)$$

The experimentally obtained quantity of H_2CID^{III} was obtained by weighing, following the purification of the final product. On the other hand, the maximum possible quantity of H_2CID^{III} represents the theoretical yield, calculated based on stoichiometry. This calculation relies on the balanced chemical equation for the reaction:

$$(\mathrm{ArBr_2} + 2\mathrm{CuCN} \rightarrow \mathrm{ArCN_2} + 2\mathrm{CuBr}) \qquad (2)$$

The excess reagent in the reaction is CuCN, and the yield was calculated based on limiting reagent, $ArBr_2$.

$^1H/^{13}C$ NMR data were used to confirm the successful synthesis of H_2CID^{III}.

^1H NMR of the dinitrile product (400 MHz, DMSO) δ 8.51 (d, J = 1.2 Hz, 1H), 8.39 (dd, J = 7.3, 1.2 Hz, 2H), 7.85 (dd, J = 8.4, 7.3 Hz, 2H). ^{13}C NMR (101 MHz, DMSO) δ 139.02, 135.76, 133.46, 128.29, 127.58, 117.16, 107.48 (Supplementary Fig. 5a and 6a). Analytic calculation for $C_{12}H_6N_2$: C, 80.88; H, 3.39; N, 15.72. Found: C, 80.10; H, 3.26; N, 15.62.

^1H NMR of H_2CID^{III} (400 MHz, DMSO) δ 11.06 (s, 1H), 8.97 (s, 0H), 8.15 (dd, J = 7.4, 1.1 Hz, 1H), 8.07 (dd, J = 8.5, 1.1 Hz, 1H), 7.64 (dd, J = 8.3, 7.3 Hz, 1H). ^{13}C NMR (101 MHz, DMSO) δ 141.53, 132.92, 129.94, 126.92, 126.17, 122.41, 121.10 (Supplementary Fig. 5b and 6b). Analytic calculation for $C_{12}H_9N_3O_2$: C, 61.76; H, 5.18; N, 15.43. Found: C, 61.75; H, 5.48; N, 15.63.

Furthermore, the chemical structure of H_2CID^{III} was characterized using fourier transform infrared (FT-IR) spectroscopy and compared to that of the dinitrile intermediate. In the FT-IR spectra (Supplementary Fig. 7), a prominent stretching vibration peak corresponding to the -C≡N group (at approximately 2226 cm^{-1}) was observed in the dinitrile intermediate. However, this peak completely disappeared in the spectrum of H_2CID^{III}, indicating the successful hydroxylamination process and conversion of the nitrile group to the oxime functionality. Additionally, H_2CID^{III} exhibited broad hydrogen bonding stretches in the range of approximately 3200-2700 cm^{-1}, along with a medium peak at around 3400 cm^{-1}, corresponding to the presence of oxime and imide functionalities on the ligand. These peaks were absent in the spectrum of the dinitrile intermediate, further confirming the structural transformation during the synthesis of H_2CID^{III}.

Supplementary Discussion 2. Acid stability assessment

The evaluation of acid stability holds crucial importance in verifying the suitability of materials for repeated adsorption/desorption cycles, given that the leaching of oil sands tailings involves concentrated acids, leading to the creation of highly acidic environments. In this study, acid stability was studied to ensure the viability of materials during complexation, precipitation, and elution processes, which are vital for the economic feasibility of extraction procedures. Kang et al. elucidated that the degradation of strong vanadium-specific binding sites in H_2CID^{I} ($t_{1/2}$ = 0.9h) (Supplementary Fig. 1a) in acidic solutions arose from water's nucleophilic attack on its structure (Supplementary Fig. 1b).[2] One approach to retard this process is to reduce the charge density of the imino carbons in H_2CID^{I}. This concept led to the synthesis of a related ligand, phthalimidedioxime (H_2CID^{II}), which exhibited enhanced acid stability ($t_{1/2}$ = 147h at 25°C) due to aromatic resonance stabilization (Supplementary Fig. 1a).[2]

To evaluate the acid stability of H_2CID^{III}, the ligand was exposed to a 1M DCl aqueous solution and monitored using ^{1}H NMR. The imide and oxime hydrogens of H_2CID^{III} appeared at 8.97 and 11.06 ppm in DMSO-d_6, respectively (Supplementary Fig. 5b), but disappeared after exposure to D_2O/DCl solution due to proton exchange between the ligand and deuterated solvent (Supplementary Fig. 5c). Consequently, hydrogens on the aromatic moiety corresponding to two doublet and triplet signals at 8.06 – 8.16 ppm and 7.66 – 7.62 ppm, respectively, were utilized to track the ligand's degradation. After several months of exposure at room temperature ($t_{1/2}$ = > 1 month at 25°C), the representative peaks were well retained (star marked spectra in Supplementary Fig. 8), and higher temperatures were necessary to accelerate hydrolysis. At 80°C, three new signals corresponding to glutaric acid and glutarimide were observed after 30 minutes and became more pronounced after 2 hours (circle-marked spectra in Supplementary Fig. 8). The hydrolysis process was complete after 196 hours of exposure (triangle-marked spectra in Supplementary Fig. 8). Thus, while H_2CID^{III} followed a similar degradation pathway via a monoxime to an imide final product, it was

much more resistant than H_2CID^{II} and H_2CID^{I} to acid-catalyzed hydrolysis at room temperature.

Supplementary Discussion 3. Spectroscopic analysis of vanadium complexes

Supplementary Fig. 11 displays the ^1H NMR spectra of pristine H_2CID^{III} and the two V-CID^{III} complexes, **1** and **2**. All samples exhibited three ^1H signals (two doublets (8.06 – 8.16 ppm) and a triplet (7.65 – 7.62 ppm)), assigned to aromatic protons of the ligand. In the 1:1 V-CID^{III} complex, **1** (Supplementary Fig. 11a), the ^1H signals shift upfield relative to the free deprotonated ligand. This shift can be attributed to the shielding effect from the electron density from the opposite aromatic ring. On the other hand, in the case of the 1:2 V-CID^{III} complex, **2** (Supplementary Fig. 11b), all ^1H signals shift downfield compared to the free deprotonated ligand. The symmetrical coordination mode suggests the complete displacement of the oxo groups on V(V), forming a non-oxido complex. The downfield shift is due to the charge of V(V) significantly deshielding ligand protons. These features confirm strong interactions between H_2CID^{III} and V(V) and stable complexes. During the isotherm experiments, depending on the metal to ligand stoichiometry, two complexes were obtained with qe values of 80 mg g^{-1} and 200 mg g^{-1}, corresponding to **2** and **1**, respectively (Fig 4b).

The ESI-MS experiments (Supplementary Fig. 12a-c) performed in methanol solutions confirmed the presence of the V-CID^{III} complexes and provided insights into their structures. In the solution containing **1** (Supplementary Fig. 12b), two peaks were observed corresponding to the monomer and dimer at m/z = 322.0 and 612.9, respectively. Surprisingly, the calculated masses for the monomer and dimer of **1** were 307.3 and 598, respectively, which significantly differed from the observed masses. This discrepancy can be attributed to the incorporation of methoxide (CH_3O^-) from the electrospray solvent during the dilution and/or electrospray process. Similar behavior was observed in the ESI-MS analysis of the 1:1 complex formed from the reaction of H_2CID^{I} and V(V), where the hydroxide (OH^-) of the complex was substituted by ethoxide ($CH_2CH_3O^-$) from the electrospray solvent.[3] However, this substitution was not observed in the

solution containing **2** (Supplementary Fig. 12a), likely due to the absence of an oxido moiety in the complex. In this case, a single peak at m/z = 499.03, which matched the calculated mass of 498.9, was obtained for **2**. Therefore, the ESI-MS data confirmed the formation of two distinct complexes upon the reaction of H_2CID^{III} with V(V).

FT-IR spectra of pristine H_2CID^{III}, crystal samples of **1**, **2** and V-CID^{III} complexes obtained from complexation and precipitation experiments (H^+-form) were acquired to verify the sites for protonation in the neutralization process. As seen in the spectrum (0) in Fig. 5b, the bands at 3450 cm^{-1}, 3300 cm^{-1}, 1620 cm^{-1} and 750 cm^{-1} in pure H_2CID^{III} can be attributed to the N–H stretching vibration in imide (–C–NH–C–) and oxime (–C–NH=O) groups, O–H stretching vibration, N–H bending in imide and oxime groups, and naphthyl C–H bending, respectively. Based on the differences in these peaks after acidification, the absence of N–H and O–H in the Na^+-form **2** and **1** is observed from the disappearance of the bands at 3450 cm^{-1}, 3300 cm^{-1} and 1620 cm^{-1} from their spectra (1, 3). While the band attributed to N–H stretching, and deformation vibration can be observed in both spectra of H^+-form V-CID^{III} complexes (2, 4); the band attributed to O–H stretching vibration can only be observed in the spectra of H^+-form 1:1 V-CID^{III} complex (4). These results demonstrate that the N in the oxime group (–C=**N**–O) is the only site in the 1:2 V-CID^{III} complex that can accept a proton during neutralization, whereas in the 1:1 V-CID^{III} complex, both the N in the oxime group and the O in the V–O group can be protonated.

Supplementary Discussion 4. Kinetic studies

Beyond high capacity, it is crucial for H_2CID^{III} to rapidly capture target ions. Kinetic experiments were conducted for the complexation and precipitation steps of V(V) capture by H_2CID^{III} at concentrations of 50 mg L^{-1} and 20 mg L^{-1}, respectively, with samples analyzed at regular intervals (see methods). In the complexation experiment, the removal rate of V(V) by H_2CID^{III} reached equilibrium within 45 minutes at 50 mg L^{-1} and within 30 minutes at 20 mg L^{-1} (Supplementary Fig. 13). The experimental data were best

fit with the pseudo-second-order kinetics model, (Supplementary Table 5). Additionally, precipitation began instantaneously at both concentrations upon acidification, achieving maximum precipitation amount within 5 minutes. Interestingly, H_2CID^{III} retained a high extraction capacity of 195.4 mg/g in even more acidic solutions (~pH 1.5) (Supplementary Fig. 18 and 19). These outcomes are promising for extracting vanadium from acidic waste sources.

Supplementary Discussion 5. Regeneration and reusability

A stoichiometric extractant would be challenging economically. To release V(V) and regenerate H_2CID^{III}, thiourea was employed due to its known ability to elute strongly bound metal ions from adsorbents (see methods).[4] For the regeneration step, the V(V) concentration in the decomplexation solution was quantified via ICP-OES analysis and used to calculate the mass of V(V) in the solution. The results of the decomplexation showed that thiourea could completely elute the bound V(V) from CID^{III} (Supplementary Fig. 14) but HCl alone was not sufficient to elute V(V). This result was corroborated by ^{1}H NMR spectroscopy and that treatment with HCl acidified thiourea solutions gave pure CID^{III} as a solid residue (Supplementary Fig. 15). After decomplexation and washing, recycled H_2CID^{III} was found to retain its initial V(V) binding capacity after six cycles of extraction and decomplexation (Supplementary Fig. 16) suggesting reusability of the ligand for multiple extraction processes.

Supplementary Figures and Tables

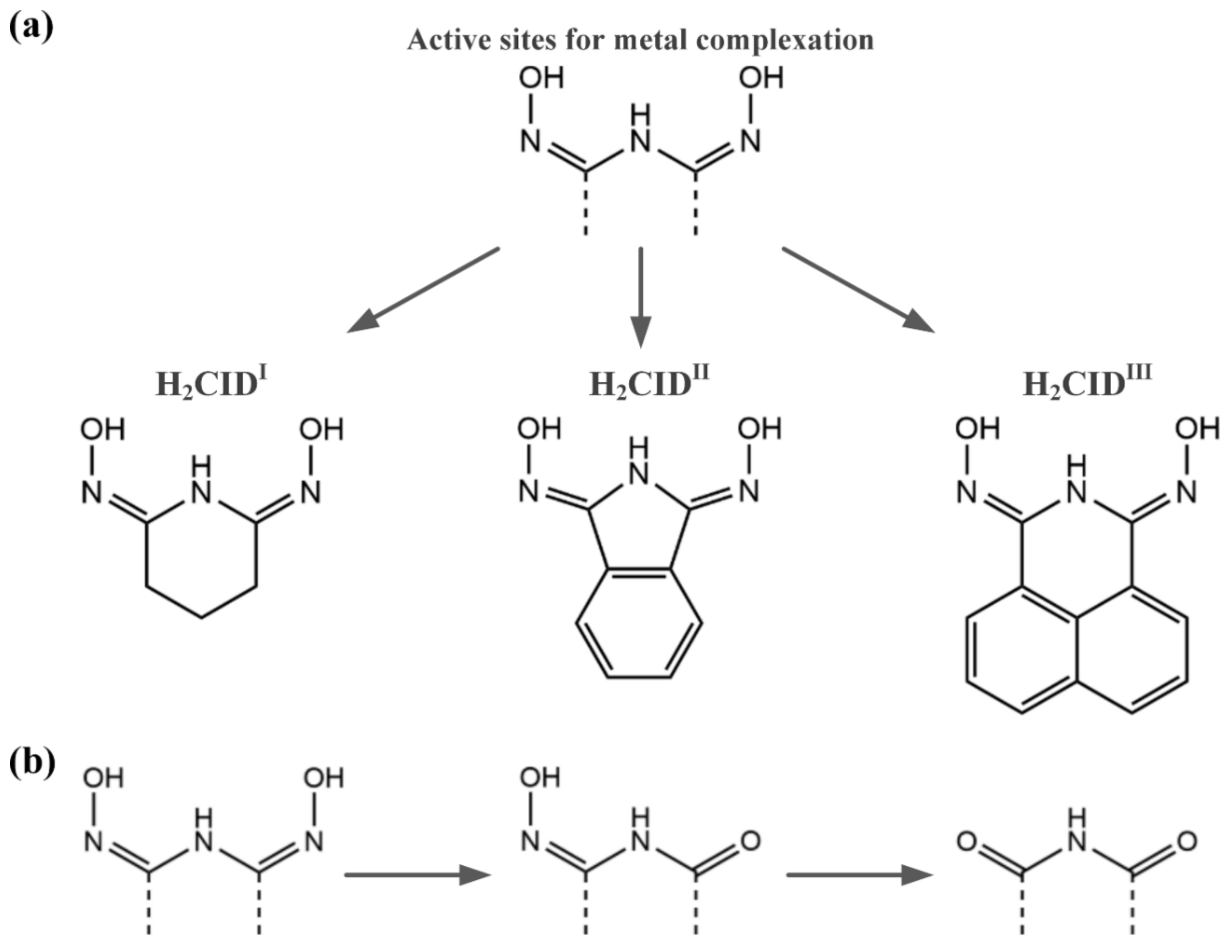

Supplementary Figure 1. Schematic representation of CyclicImideDioximes (CIDs) and their degradation. a Structures of glutarimidedioxime (H_2CID^{I}), phthalimidedioxime (H_2CID^{II}) and napthalimidedioxime (H_2CID^{III}). **b** The degradation pathway of metal-specific binding sites.

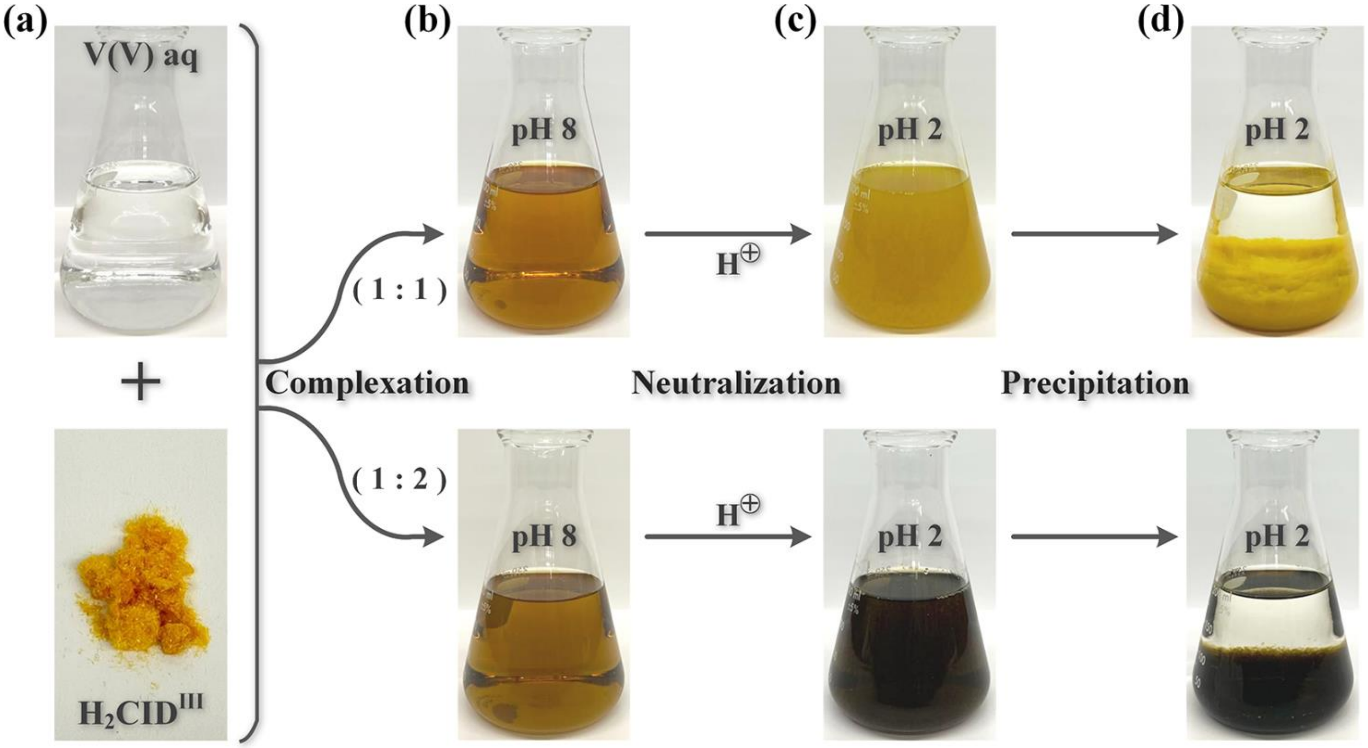

Supplementary Figure 2. Complexation-precipitation technique for recovery of vanadium. a V(V) aqueous solution and H_2CID^{III}. **b** V-CID^{III} complexes in solution. **c** pH-adjusted (8 to 2) solutions with V-CID^{III} complex precipitates **d** settled V-CID^{III} precipitates in pH-adjusted solutions.

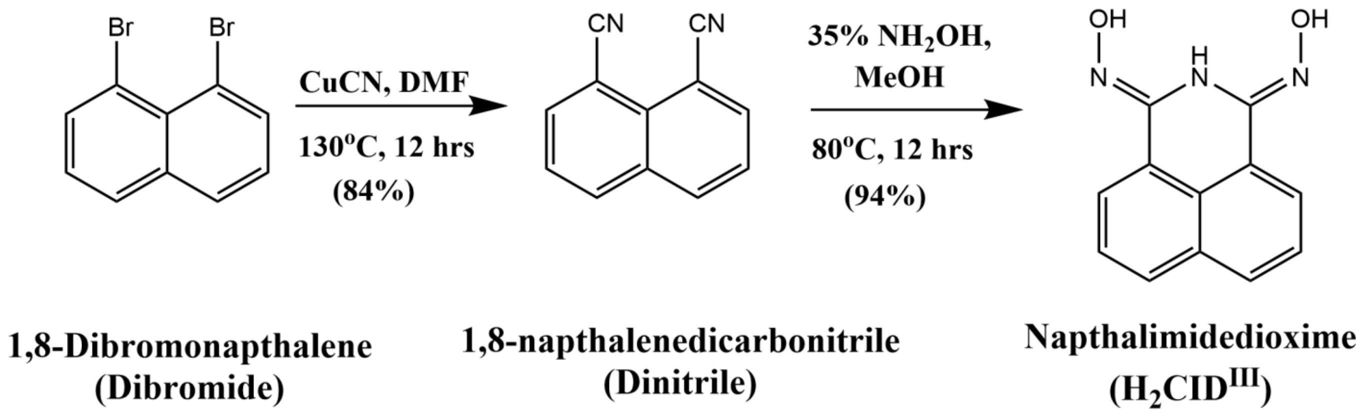

Supplementary Figure 3. Improved synthetic route for H_2CID^{III}.

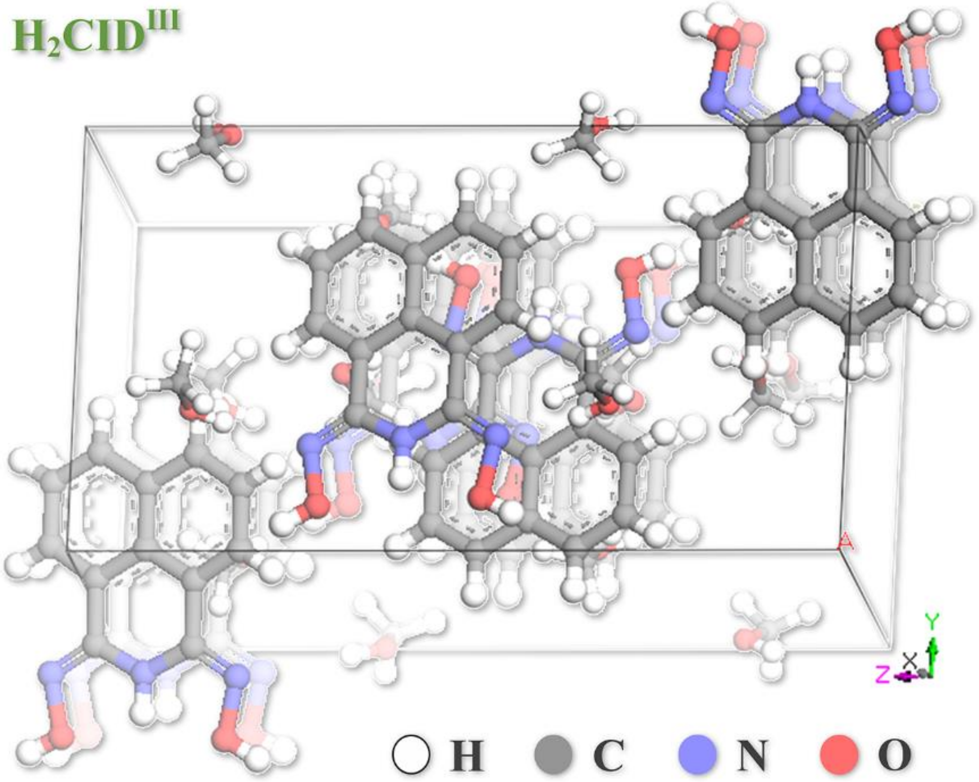

Supplementary Figure 4. Unit cell configuration of H_2CID^{III}

Supplementary Table 1. Crystallographic data and structure refinement of H_2CID^{III}

**Identification code**	**H_2CID^{III}**
Emperical formula	$C_{14}H_{17}N_3O_4$
Formular weight	291.30
Temperature/K	173.0
Crystal system	Monoclinic
Space group	$P2_1/n$
a/Å	17.654(6)
b/Å	9.529(3)
c/Å	18.411(6)
α/°	90
β/°	106.840(5)
γ/°	90
Volume/$Å^3$	2964.4(17)
Z	8
$\rho_{calc}g/cm^3$	1.305
μ/mm^{-1}	0.097
F(000)	1232.0
Crystal size/mm^3	0.457 × 0.405 × 0.142
Radiation	MoKα (λ = 0.71073)
2Θ range for data collection/°	2.814 to 56.576
Index ranges	$-23 \le h \le 22, -12 \le k \le 12, -24 \le l \le 24$
Reflections collected	56723
Independent reflections	7371 [R_{int} = 0.0573, R_{sigma} = 0.0335]
Data/restraints/parameters	7371/0/413
Goodness-of-fit on F^2	1.028
Final R indexes [I>=2σ (I)]	R_1 = 0.0525, wR_2 = 0.1371
Final *R* indices [all data]	R_1 = 0.0946, wR_2 = 0.1601
Largest diff. peak/ hole/ e $Å^{-3}$	0.34/-0.24

(a)

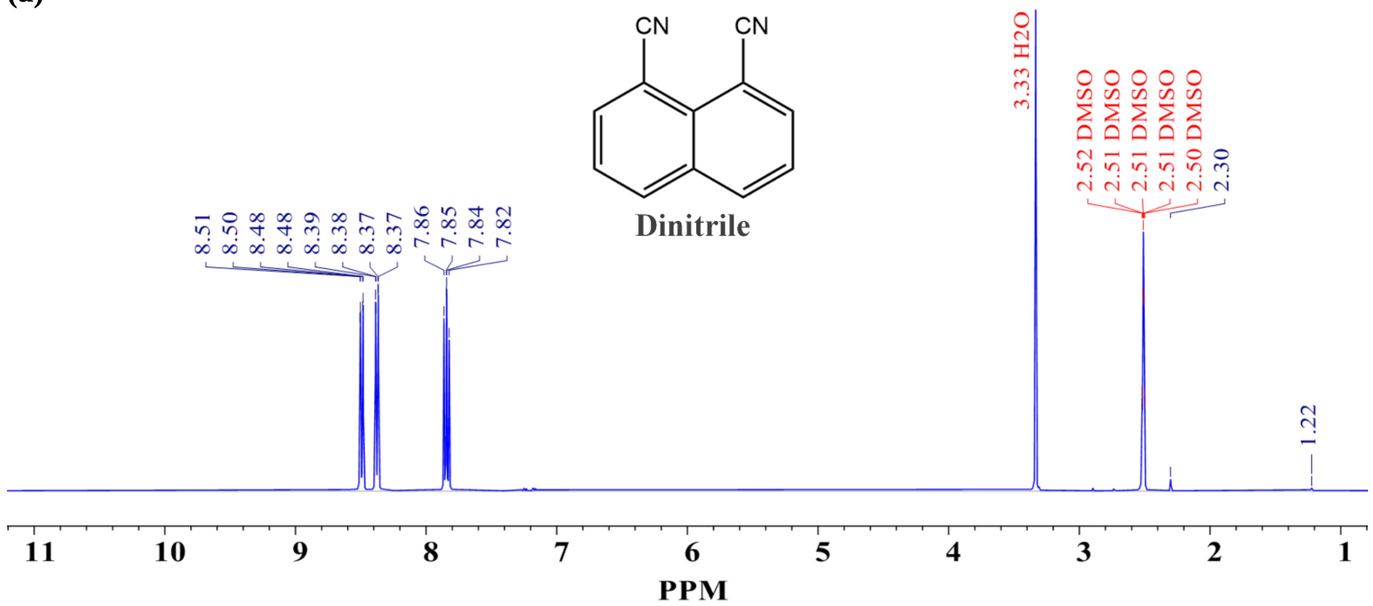
CN
CN
Dinitrile
8.51
8.50
8.48
8.48
8.39
8.38
8.37
8.37
7.86
7.85
7.84
7.82
3.33 H2O
2.52 DMSO
2.51 DMSO
2.51 DMSO
2.51 DMSO
2.50 DMSO
2.30
1.22
11
10
9
8
7
6
5
4
3
2
1
PPM

(b)

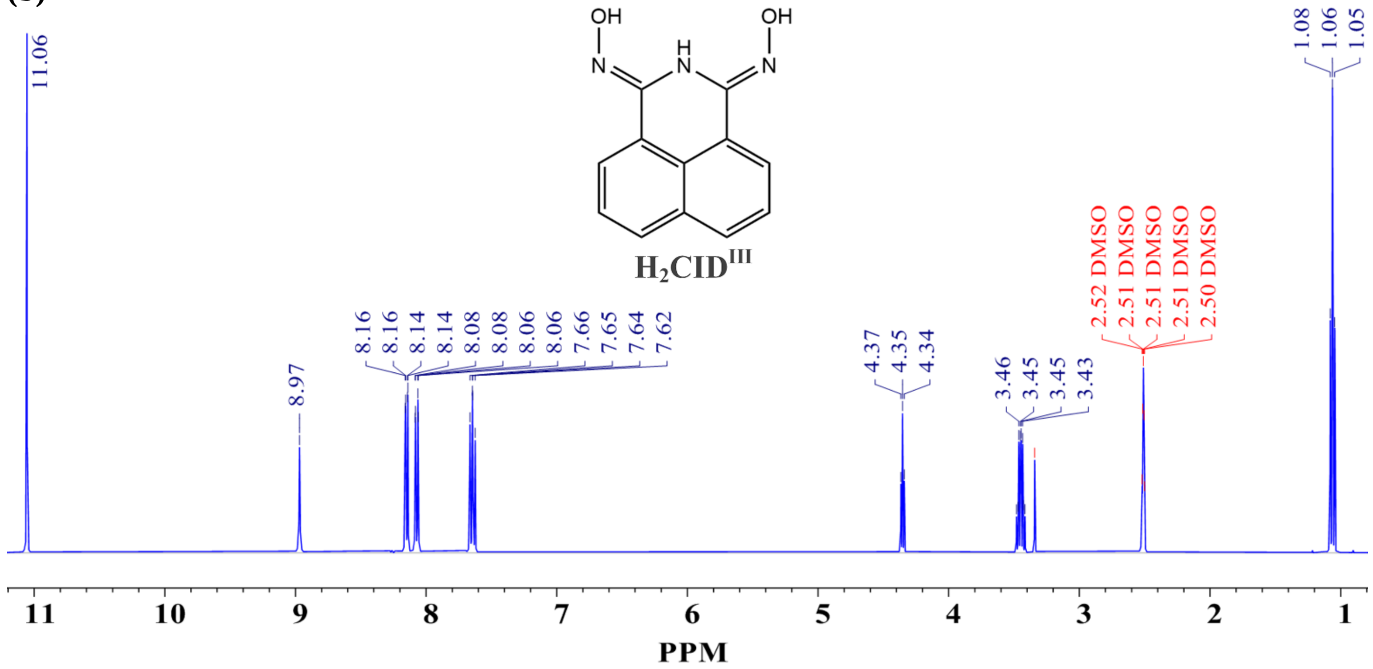
OH
OH
N
H
N
N
H_2CID^{III}
11.06
8.97
8.16
8.16
8.14
8.14
8.08
8.08
8.06
8.06
7.66
7.65
7.64
7.62
4.37
4.35
4.34
3.46
3.45
3.45
3.43
2.52 DMSO
2.51 DMSO
2.51 DMSO
2.51 DMSO
2.50 DMSO
1.08
1.06
1.05
11
10
9
8
7
6
5
4
3
2
1
PPM

(c)

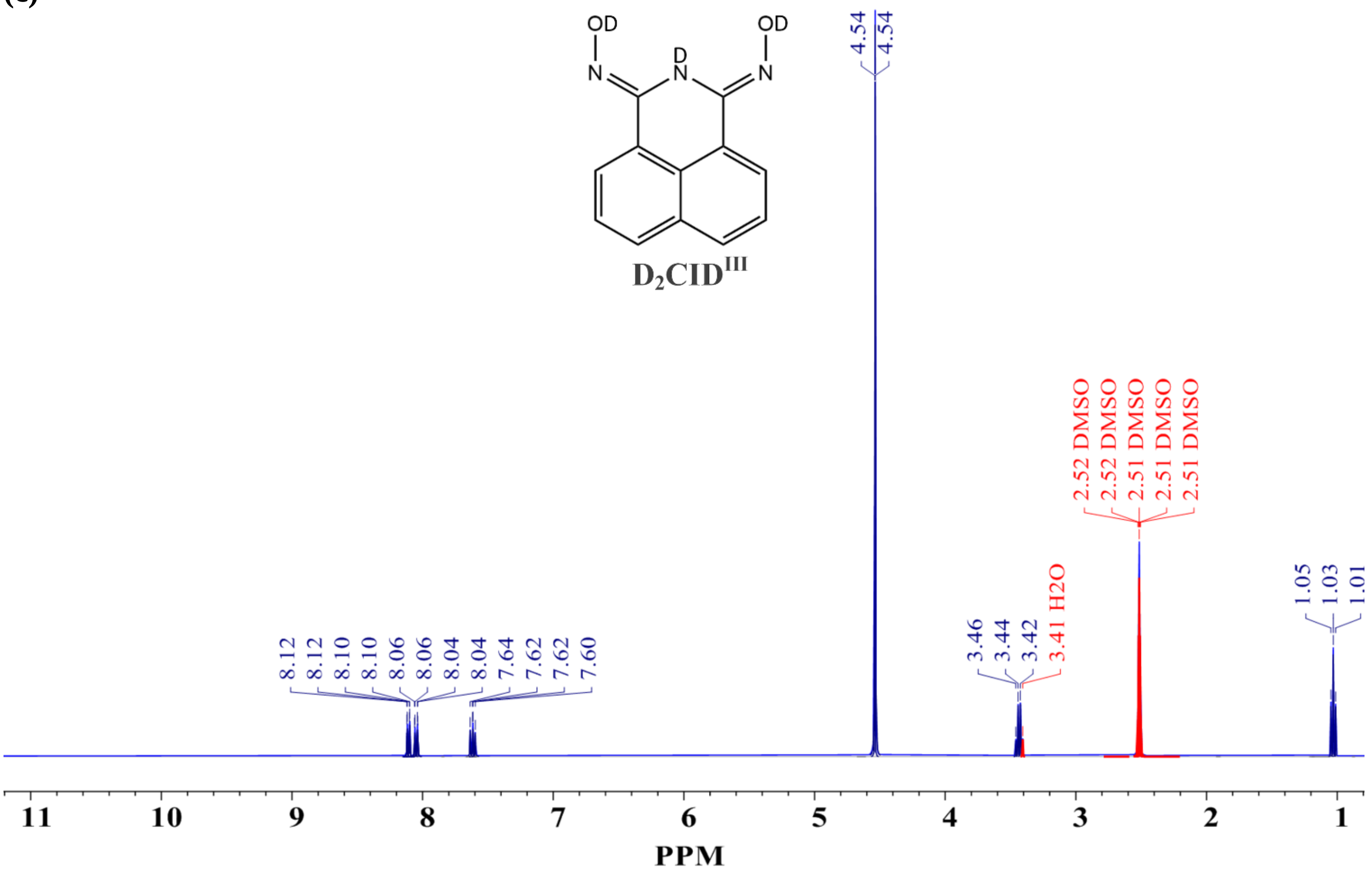

Supplementary Figure 5. ^{1}H NMR spectra. a dinitrile**. b** H_2CID^{III}. **c** D_2CID^{III}.

(a)

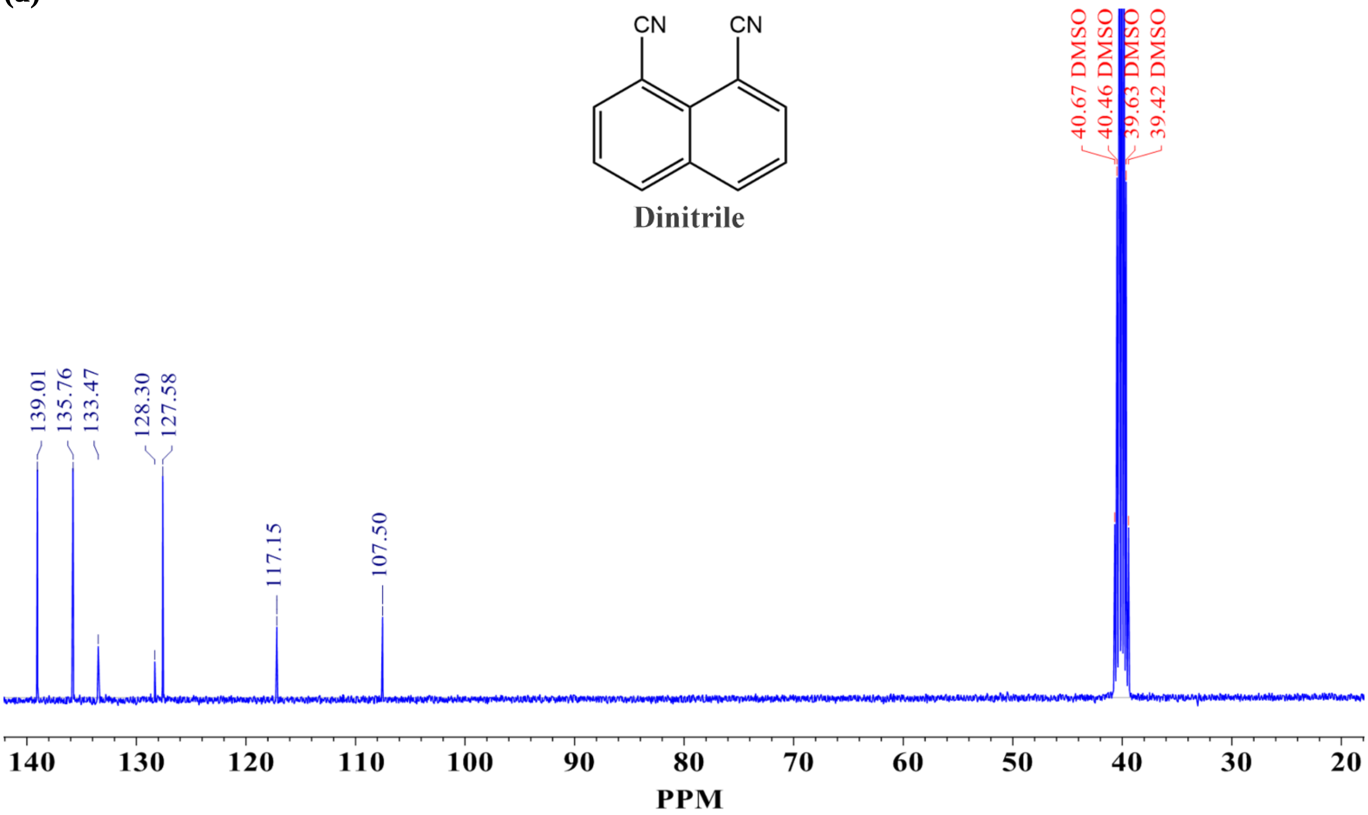

(b)

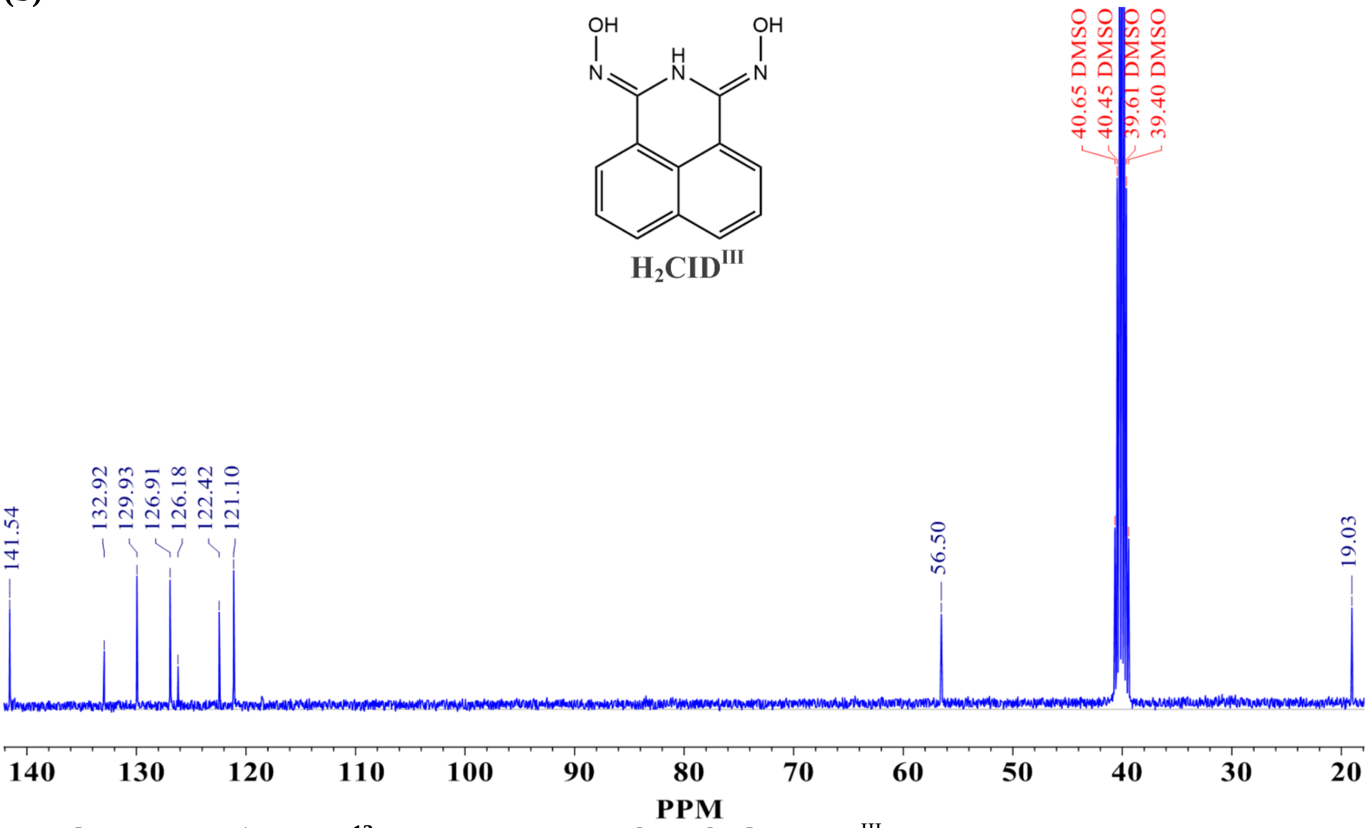

Supplementary Figure 6. ^{13}C NMR spectra. a dinitrile**. b** H_2CID^{III}.

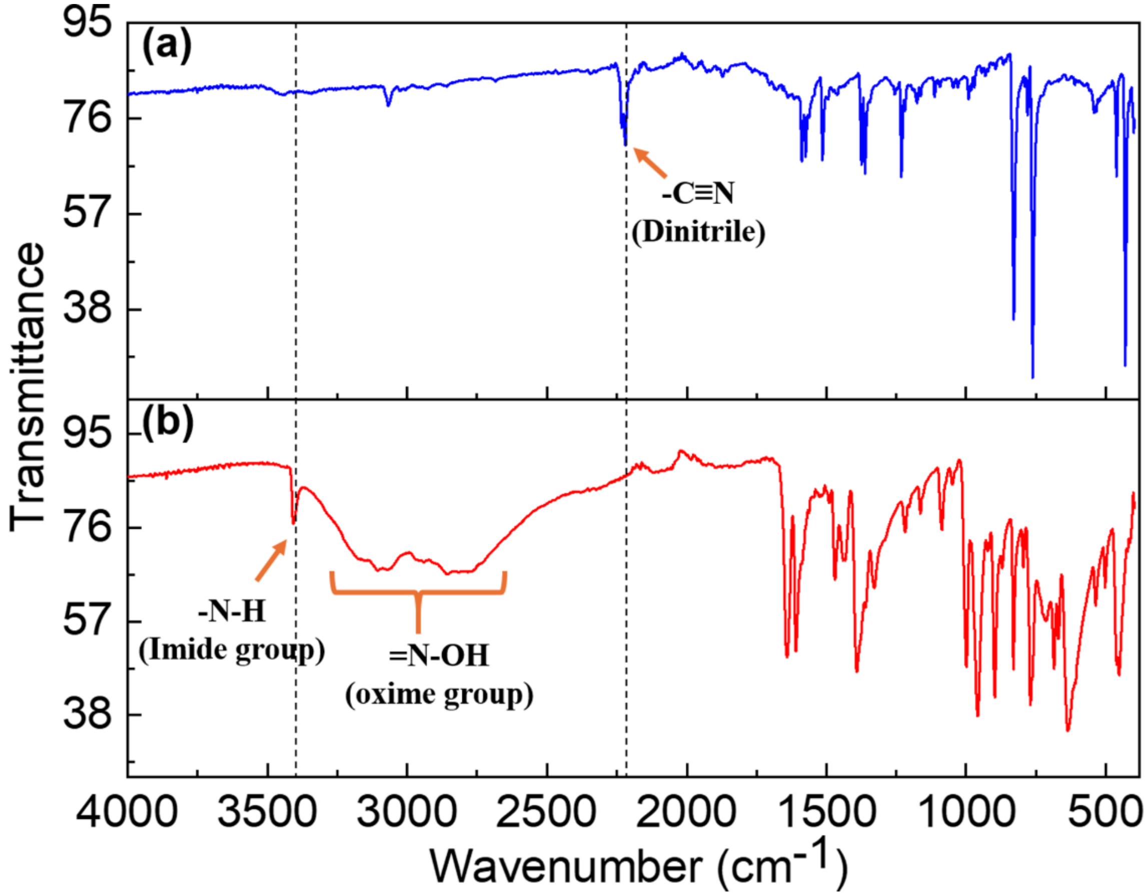

Supplementary Figure 7. FTIR of synthesis products. **a** Dinitrile. **b** H_2CID^{III}.

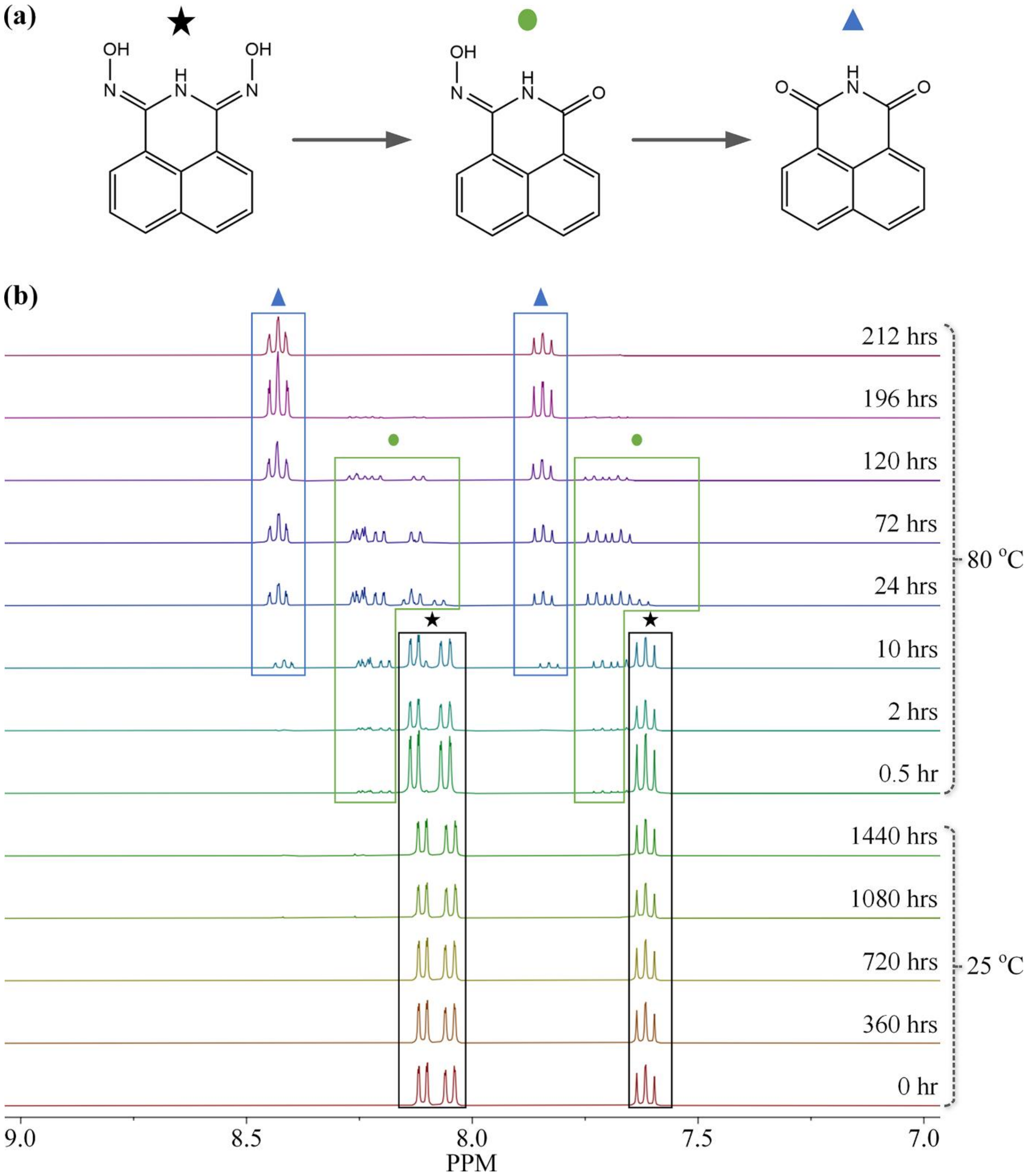

Supplementary Figure 8. Evolution of the ^{1}H NMR spectrum and degradation pathways of H_2CID^{III}.

a The degradation pathway of H_2CID^{III}. **b** Evolution of the ^{1}H NMR spectrum of H_2CID^{III} with time in 1 M DCl at 25°C and 80°C ($t_{1/2}$ = > 1 month and 196 hours, respectively).

Supplementary Table 2. Crystallographic data and structure refinement of 1:1 V-CID^{III} complex, 1.

**Identification code**	**1:1 V-CID^{III}**
Emperical formula	$C_{33}H_{30}N_6Na_2O_{10}V_2$
Formular weight	818.49
Temperature/K	173
Crystal system	Monoclinic
Space group	$P2_1/n$
a/Å	7.8419(2)
b/Å	28.2476(7)
c/Å	15.9108(4)
α/°	90
β/°	92.898(2)
γ/°	90
Volume/Å^3	3519.97(15)
Z	4
ρ_{calc}g/cm^3	1.544
μ/mm^{-1}	5.266
F(000)	1672.0
Crystal size/mm^3	0.318 × 0.071 × 0.048
Radiation	CuKα (λ = 1.54178)
2Θ range for data collection/°	6.258 to 133.28
Index ranges	$-7 \leq h \leq 9$, $-32 \leq k \leq 33$, $-17 \leq l \leq 18$
Reflections collected	32011
Independent reflections	6069 [R_{int} = 0.0442, R_{sigma} = 0.0314]
Data/restraints/parameters	6069/0/484
Goodness-of-fit on F^2	1.037
Final R indexes [I>=2σ (I)]	R_1 = 0.0309, wR_2 = 0.0770
Final *R* indices [all data]	R_1 = 0.0379, wR_2 = 0.0803
Largest diff. peak/ hole/ e Å^{-3}	0.33/-0.25

Supplementary Table 3. Crystallographic data and structure refinement of 1:2 V-CID^{III} complex, 2.

**Identification code**	**1:2 V-CID^{III}**
Emperical formula	$C_{31}H_{28}N_6NaO_6V$
Formular weight	654.52
Temperature/K	173.0
Crystal system	Triclinic
Space group	P-1
a/Å	8.4698(3)
b/Å	12.0186(6)
c/Å	15.1203(6)
α/°	103.702(3)
β/°	103.305(4)
γ/°	94.079(4)
Volume/Å^3	1442.32(11)
Z	2
ρ_{calc}g/cm^3	1.507
μ/mm^{-1}	3.509
F(000)	676.0
Crystal size/mm^3	0.212 × 0.06 × 0.046
Radiation	CuKα (λ = 1.54178)
2Θ range for data collection/°	6.222 to 136.584
Index ranges	-10 ≤ h ≤ 10, -14 ≤ k ≤ 14, -18 ≤ l ≤ 18
Reflections collected	42023
Independent reflections	5088 [R_{int} = 0.0311, R_{sigma} = 0.0160]
Data/restraints/parameters	5088/0/410
Goodness-of-fit on F^2	1.054
Final R indexes [I>=2σ (I)]	R_1 = 0.0311, wR_2 = 0.0875
Final *R* indices [all data]	R_1 = 0.0327, wR_2 = 0.0891
Largest diff. peak/ hole/ e Å^{-3}	0.52/-0.26

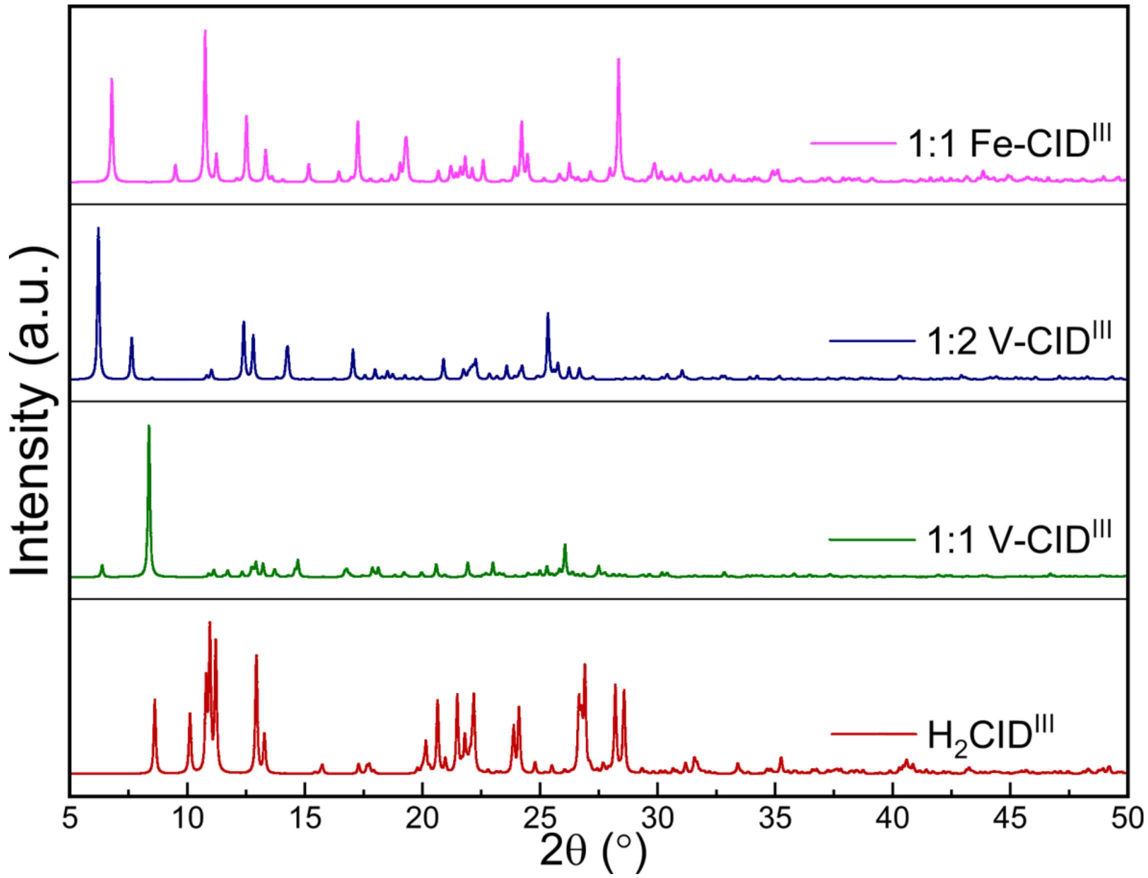

Supplementary Figure 9. XRD patterns of ligand and complexes obtained from single crystal.

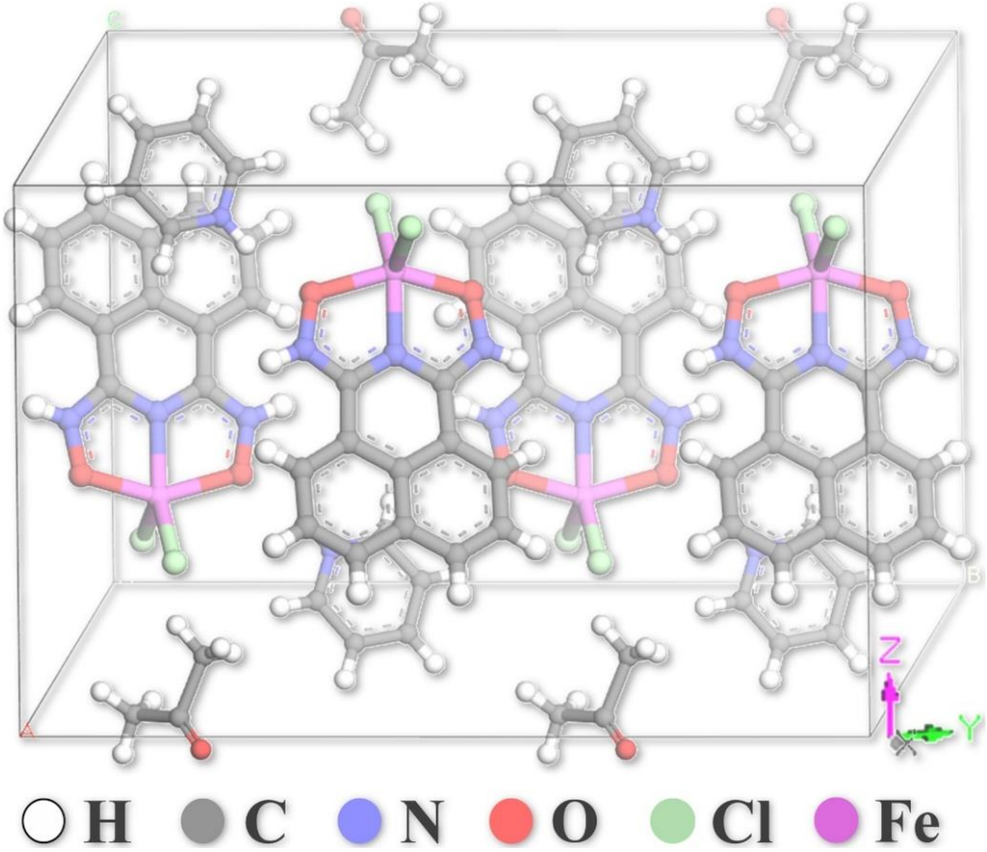

Supplementary Figure 10. Unit cell configuration of 1:1 Fe-CID^{III} complex.

Supplementary Table 4. Crystallographic data and structure refinement of 1:1 Fe-CID^{III} complex.

**Identification code**	**1:1 Fe-CID^{III}**
Emperical formula	$C_{20}H_{20}Cl_3FeN_4O_3$
Formular weight	526.60
Temperature/K	173.0
Crystal system	Triclinic
Space group	P-1
a/Å	9.3427(3)
b/Å	10.3852(3)
c/Å	13.4374(4)
α/°	93.163(2)
β/°	101.365(2)
γ/°	114.877(2)
Volume/Å^3	1145.55(6)
Z	2
ρ_{calc}g/cm^3	1.527
μ/mm^{-1}	8.753
F(000)	538.0
Crystal size/mm^3	0.355 × 0.077 × 0.07
Radiation	CuKα (λ = 1.54178)
2Θ range for data collection/°	6.792 to 133.166
Index ranges	-10 ≤ h ≤ 11, -12 ≤ k ≤ 12, -15 ≤ l ≤ 15
Reflections collected	13319
Independent reflections	3902[R_{int} = 0.0518, R_{sigma} = 0.0512]
Data/restraints/parameters	3902/181/321
Goodness-of-fit on F^2	1.047
Final R indexes [I>=2σ (I)]	R_1 = 0.0311, wR_2 = 0.0875
Final *R* indices [all data]	R_1 = 0.0327, wR_2 = 0.0891
Largest diff. peak/ hole/ e Å^{-3}	0.52/-0.26

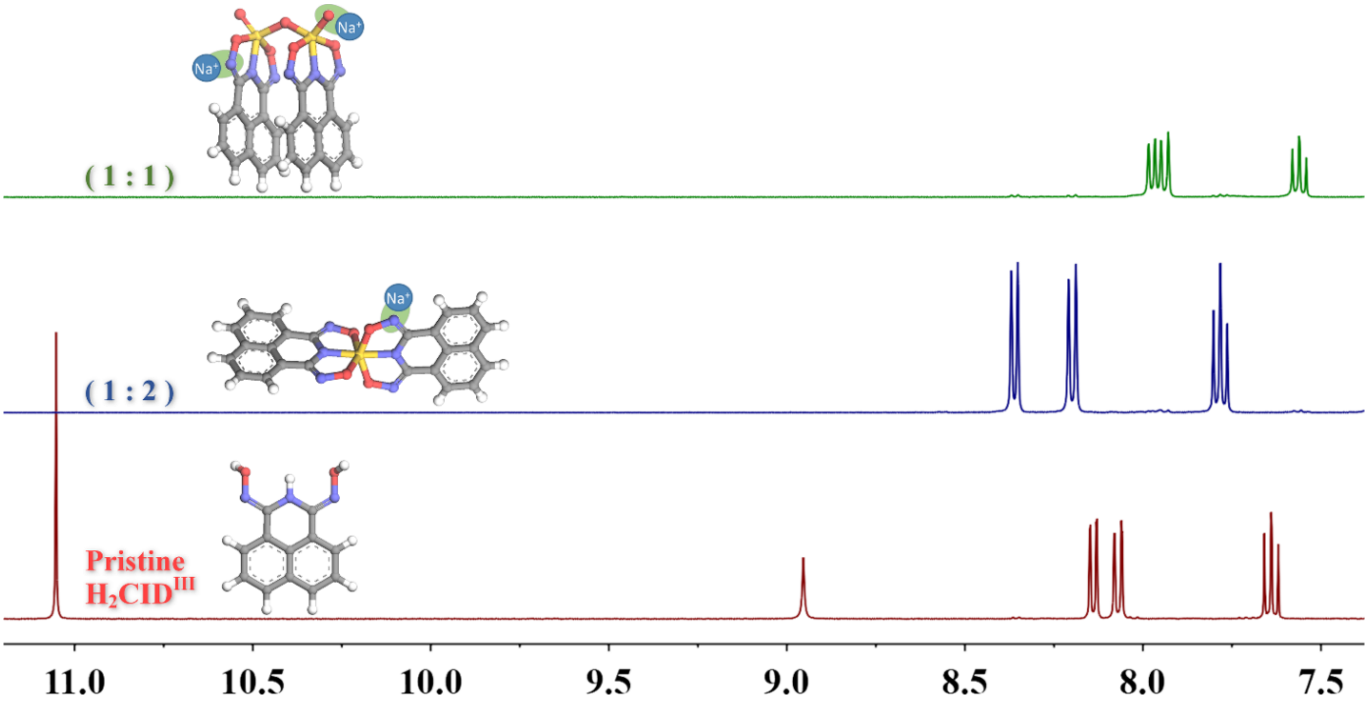

Supplementary Figure 11. **^{1}H NMR spectra of H2CID^{III} and V-CID^{III} complexes.**

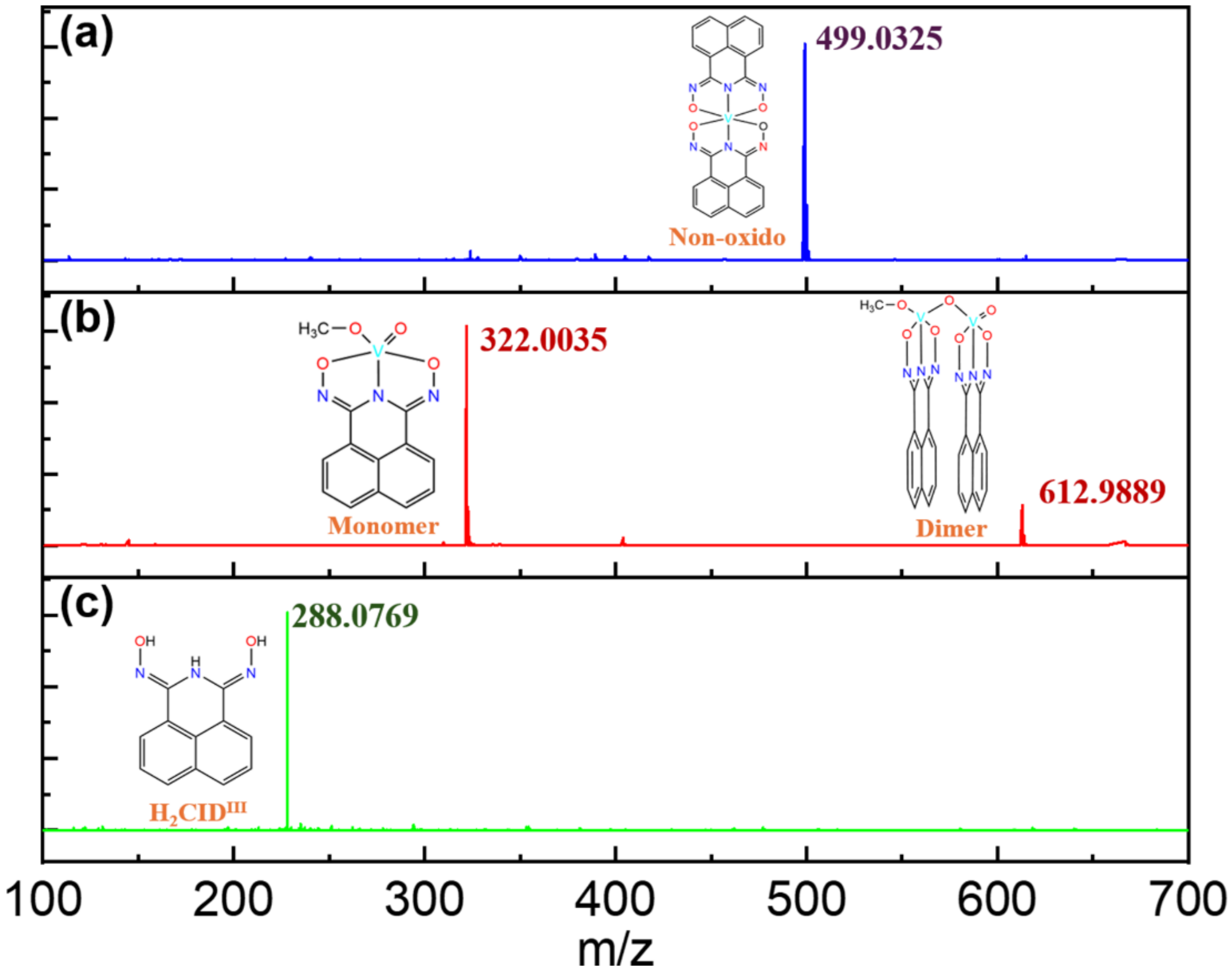

Supplementary Figure 12. ESI-MS spectra of H_2CID^{III} and V-CID^{III} complexes. a V-CID^{III} 1:2, **2 b** V-CID^{III} 1:1, **1** monomer and dimer **c** H_2CID^{III}.

Supplementary Table 5. Isotherm and kinetic parameters of the Langmuir and pseudo-second-order model, respectively.

Method	Initial Concentration	Langmuir model [a]		
		q_m (mg g^{-1})	*b* (L mg^{-1})	R^2
Two-step [b]	5 to 200 mg L^{-1} (Complexation)	205.4	2.71	0.963
One-step [c]	5 to 200 mg L^{-1} (Recovery)	195.8	3.70	0.986
**Method**	**Initial Concentration**	**Pseudo-second-order model [d]**		
		q_e (mg g^{-1})	k_2 (g mg^{-1} min)	R^2
Two-step	50 mg L^{-1} (Complexation)	204.8	0.057	0.985
	20 mg L^{-1} (Complexation)	90.0	0.066	0.959
	50 mg L^{-1} (Precipitation)	203.3	1.491	0.999
	20 mg L^{-1} (Precipitation)	83.4	2.516	0.999
One-step	50 mg L^{-1} (Recovery)	216.6	0.007	0.988
	20 mg L^{-1} (Recovery)	100.1	0.010	0.962

[a] Langmuir model: $q_e = bq_mC_e/(1 + bC_e)$, while C_e is the equilibrium concentration (mg L^{-1}) of V(V) in the solution; q_e is the equilibrium extraction amount (mg g^{-1}); *b* is the Langmuir constant related to the affinity between the V(V) and H_2CID^{III}; and the q_m is the maximum extraction capacity.

[b] Complexation at pH 8, and precipitation at pH 2.

[c] Complexation and precipitation combined as one step at pH 1.5.

[d] Pseudo-second-order model: $q_t = q_e^2k_2t/(1 + q_ek_2t)$, while q_e is the V(V) extraction amount at equilibrium, q_t is the extraction amount at any time (mg g^{-1}), and k_2 is the pseudo-second-order rate constants.

Supplementary Table 6. Comparison of adsorbent/material performance in the recovery of V(V) from several sources.

Technology	Material	ID	V(V) source	Capacity (mg g^{-1})	b value (L mg^{-1})	Ref.
Complexation-precipitation	Napthalimidedioxime	H_2CID^{III}	Oil sands tailings	205.4	2.71	This work
Adsorption	Inorganic-biopolymer composite	chitosan-zirconium (IV)	Simulated solutions	208	1.68	5
Adsorption	Commercial iron sorbent	CFH-12	synthesis gas scrubber	7.4	1.41	6
Ion exchange	Thio functionalized layered double hydroxide	S_2O_4 LDH	Spiked lake, tap and pond water	112.3	1.264	7
Ion exchange	Sawdust	TEA-I-SD	Mine water	31.6	0.835	8
Adsorption	Chitosan bead modified with titanium ions	TiCB	Simulated solutions	210	0.48	9
Adsorption	Sulfonated calixarene modified Poly (methyl methacrylate) nanoparticles	C-PMN	Spiked well, lake and tap water	322.6	0.477	10
Adsorption	Resin 201*7	Resin 201*7	Waste liquid	48.7	0.246	11
Adsorption	Red mud modified sawdust biochar	RM-BC	Simulated solutions	16.45	0.202	12
Adsorption	Nanoporous carbon materials (carbon nanotubes/MOF-199)	NPC@CNT	Simulated solutions	285.7	0.18	13
Ion Exchange	Cellulose-based anion exchanger (Amine-Modified Poly (glycidyl methacrylate)-Grafted Cellulose)	Cell-AE	Simulated solutions	197.75	0.175	14
Adsorption	KOH modified seaweed (Ascophyllum nodosum)	HC_{KOH}	Simulated solutions	12.33	0.166	15
Ion exchange	Nanosized hydrous zirconium oxide (HZrO) on anion exchange resin D201	HZrO@D201	Contaminated groundwater	110.9	0.1529	16
Adsorption	Carbon quantum dots decorated-polymeric nanocomposite	CQDots@PHQFB	Wastewater	210	0.149	17
Adsorption	Binary oxide (TiO_2-ZnO type)	$T7Zn_3$	Vanadium-based catalyst	195.9	0.014	18

Electrocoagulation	Zinc anode	ZnO	Simulated solutions	158.76	0.13	19
Adsorption	Cellulose nanocrystal	HDTMA-Br/CNC	Simulated solutions	37.9	0.13	20
Ion exchange	Palm fruit husk	PFCTAB	Simulated solutions	16.13	0.099	21
Adsorption	ZIF-8-derived porous carbon	ZDPC	Simulated solutions	89.85	0.01	22
Adsorption	Phosphoric acid modified rice straw (AcM)	AcM	Simulated solutions	21.3	0.008	23
Adsorption	Amidoxime resin	LSC700	Bayer liquor	48.08	0.0534	24
Adsorption	Steel slag	SS	Simulated solutions	5.446	0.0477	25
Adsorption	Magnetic chitosan nanoparticles	Fe_3O_4-CSN	Oil refinery and jewelry wastewaters	186.6	0.07	26
Ion exchange	Resin	Amberlite®IRA-400	Steel slag leachate	27	0.004	27
Adsorption	Exchange resin	SIRs-CI	Simulated solutions	107.5	0.0029	28

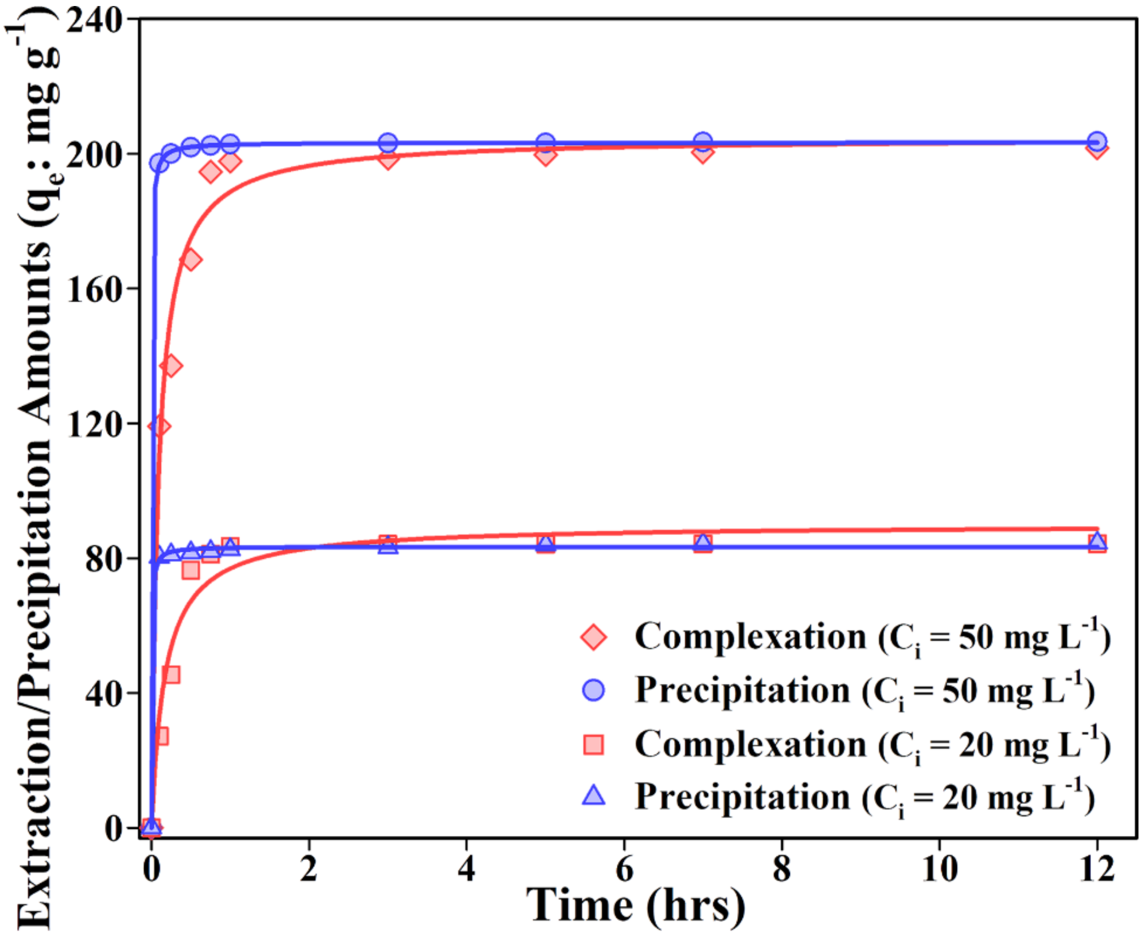

Supplementary Figure 13. The extraction kinetics of V(V) on H_2CID^{III} from aqueous solutions with initial concentrations of 20 and 50 mg L^{-1}. Source data are provided as a Source Data file.

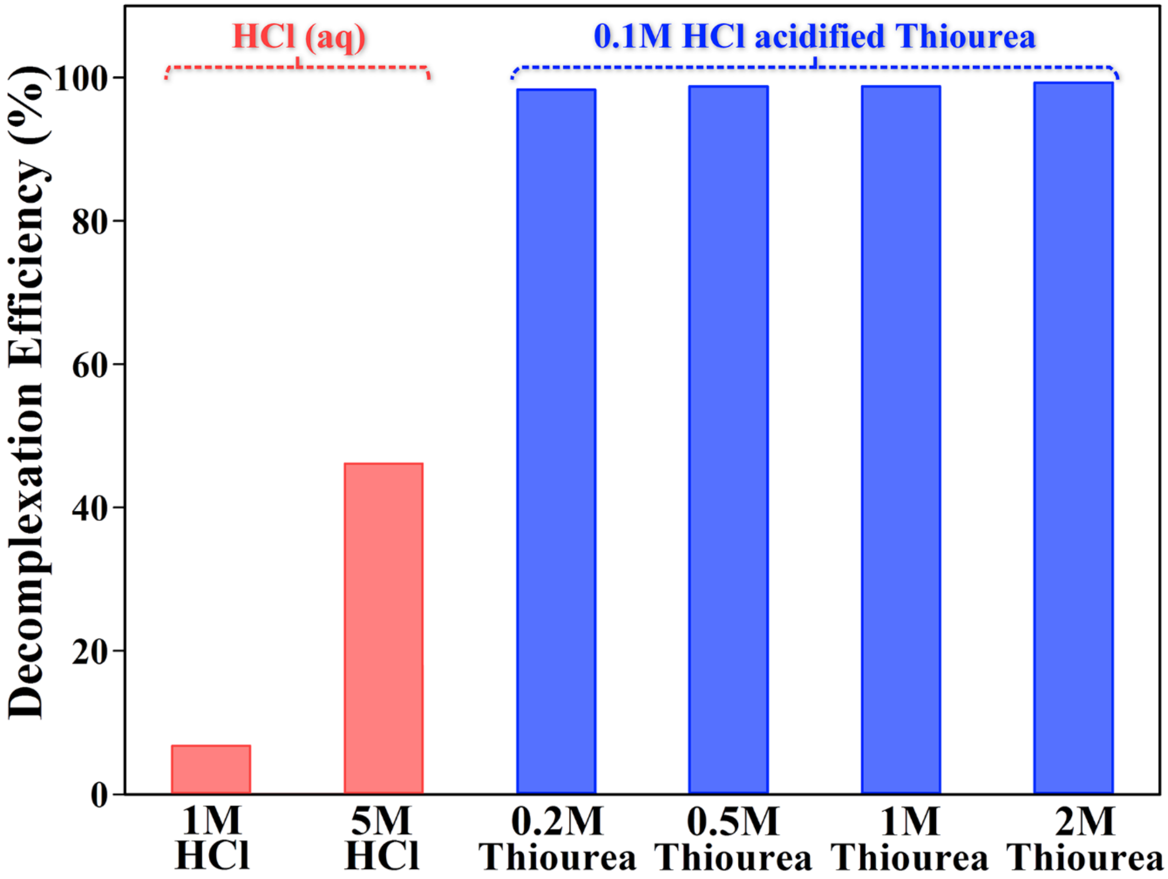

Supplementary Figure 14. The efficiencies of various reagents for decomplexing V(V) from its complex. Source data are provided as a Source Data file.

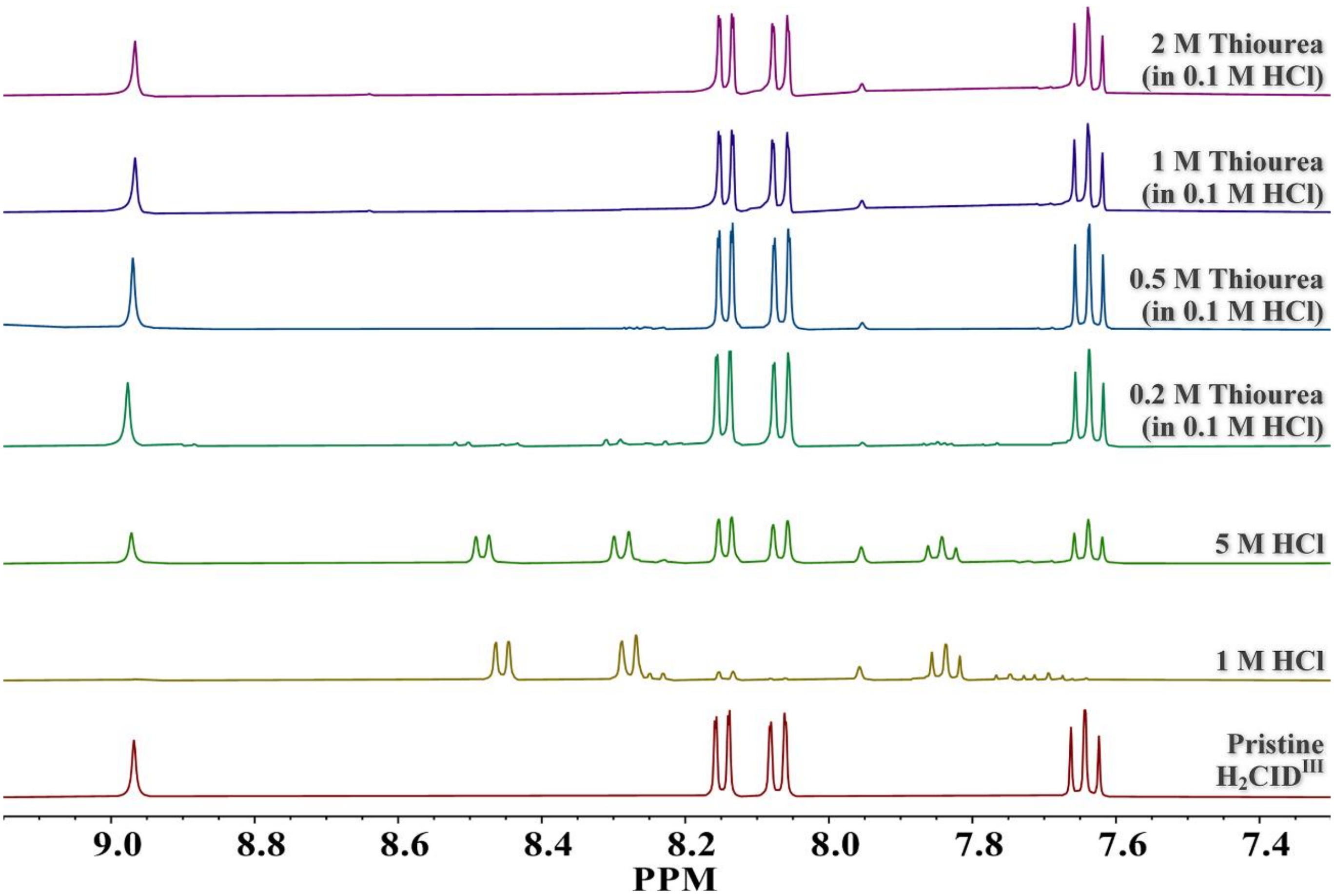

Supplementary Figure 15. **^{1}H NMR of pristine H_2CID^{III}, and V-CID^{III} complex after immersion in HCl solutions and HCl acidified thiourea solutions.**

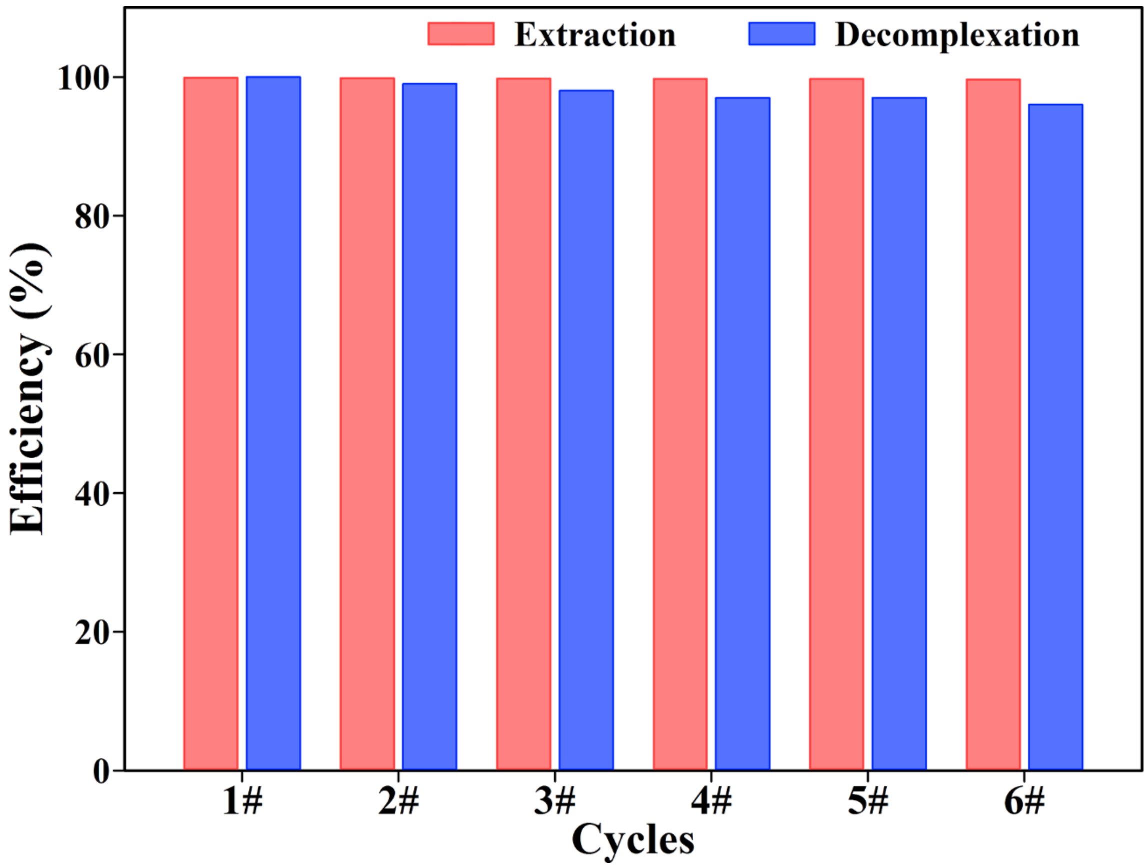

Supplementary Figure 16. Extraction and decomplexation cycles of H_2CID^{III} for V(V). Source data are provided as a Source Data file.

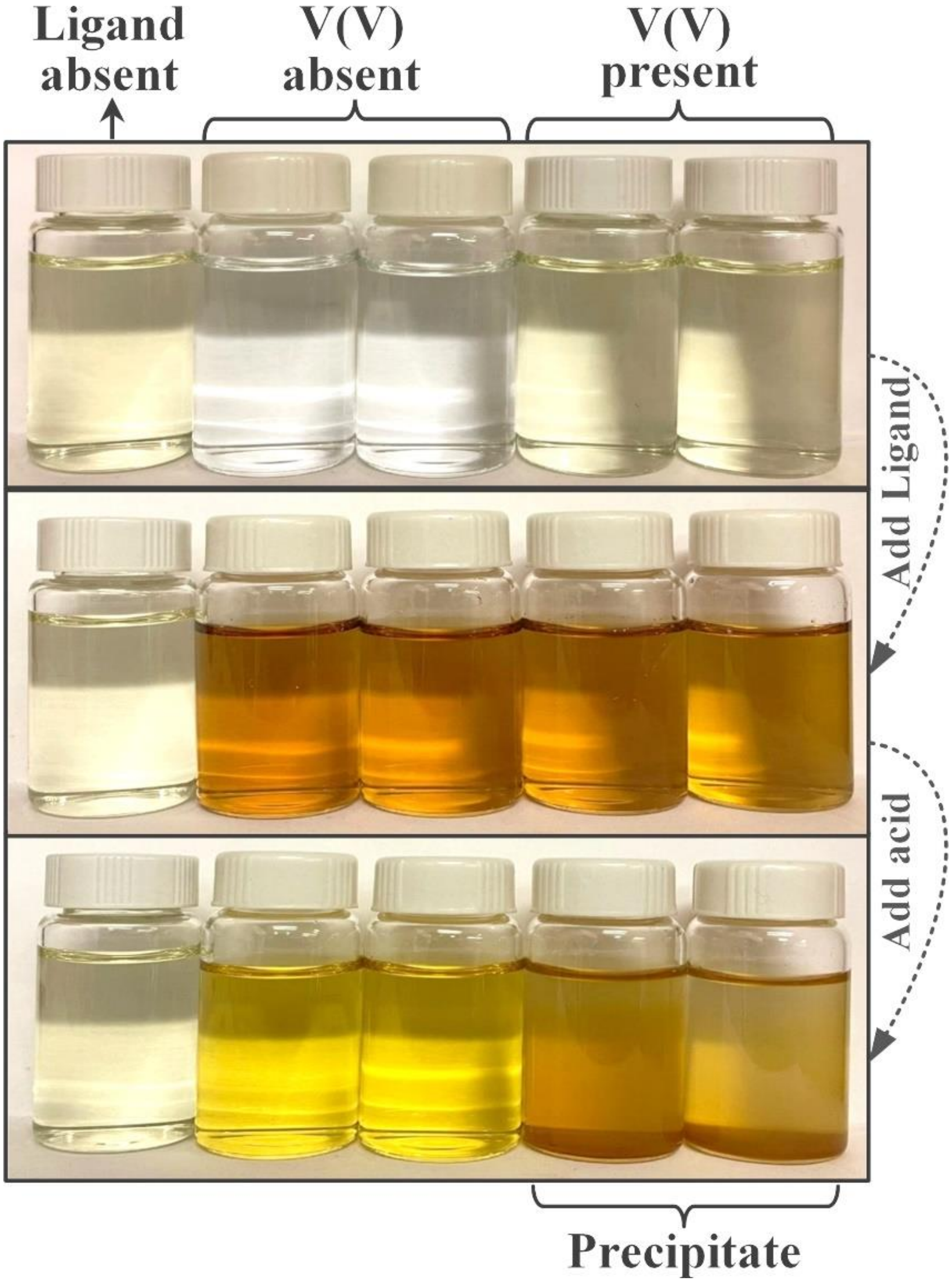

Supplementary Figure 17. Control experiments for selective recovery of V(V) from mixed metal ion solutions. Solutions contained a mixture of V(V), Fe(III), Cr(III), Cu(II), Ni(II), and Zn(II).

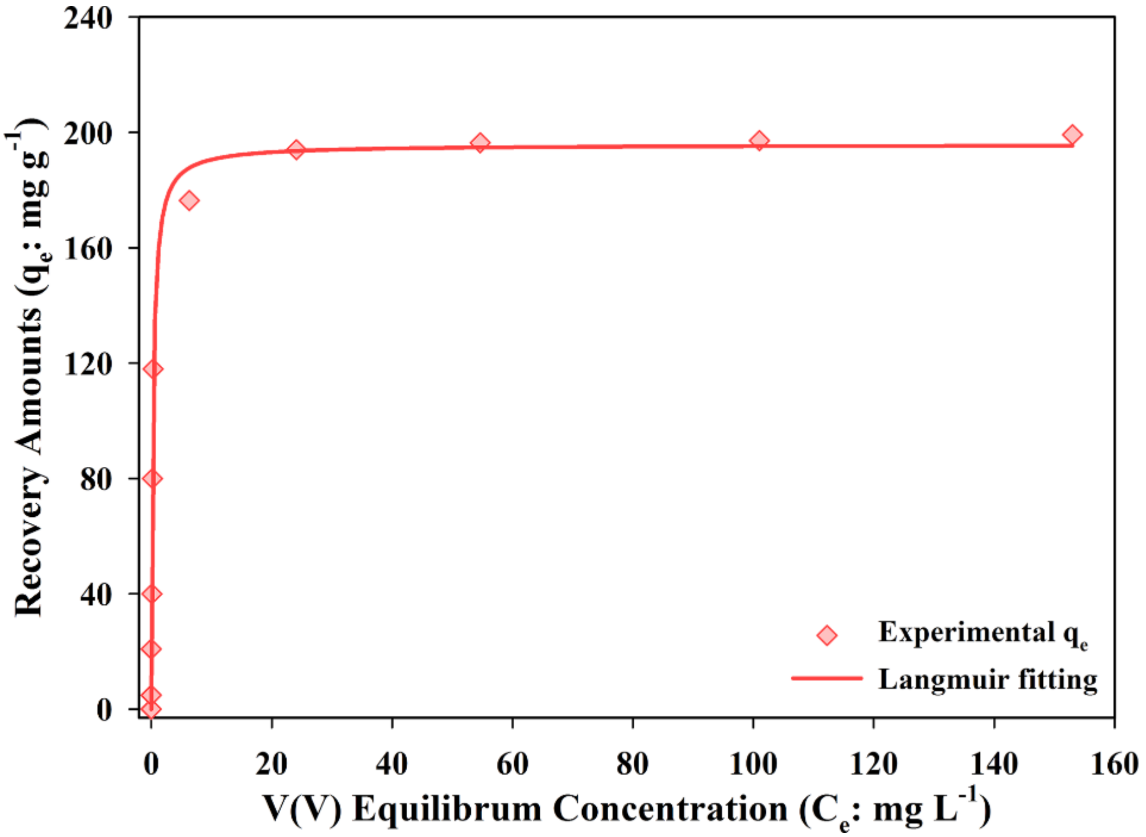

Supplementary Figure 18. The recovery isotherm of V(V) on H_2CID^{III} from aqueous solutions (at pH = 1.5) with initial concentrations of 1 to 200 mg L^{-1} via a one-step complexation and precipitation process. Source data are provided as a Source Data file.

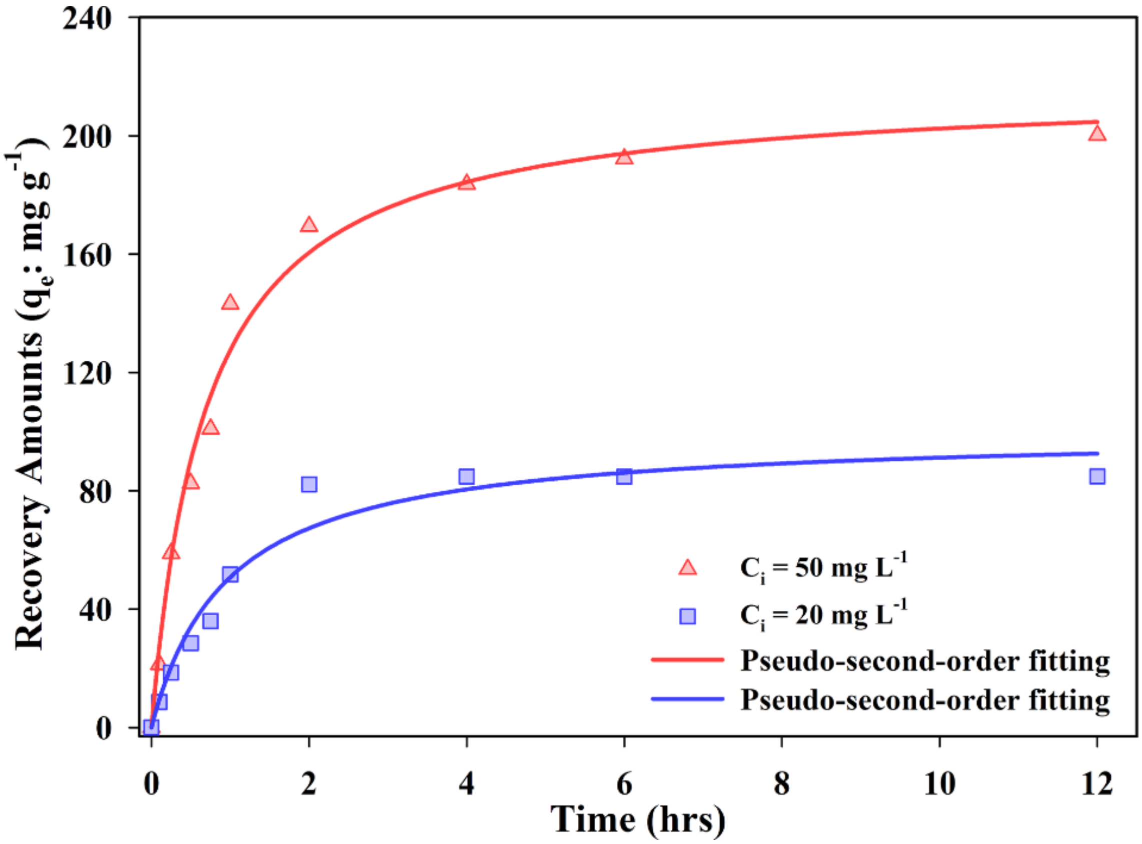

Supplementary Figure 19. The recovery kinetics of V(V) on H_2CID^{III} from aqueous solutions (at pH = 1.5) with initial concentrations of 20 and 50 mg L^{-1} via a one-step complexation and precipitation process. Source data are provided as a Source Data file.

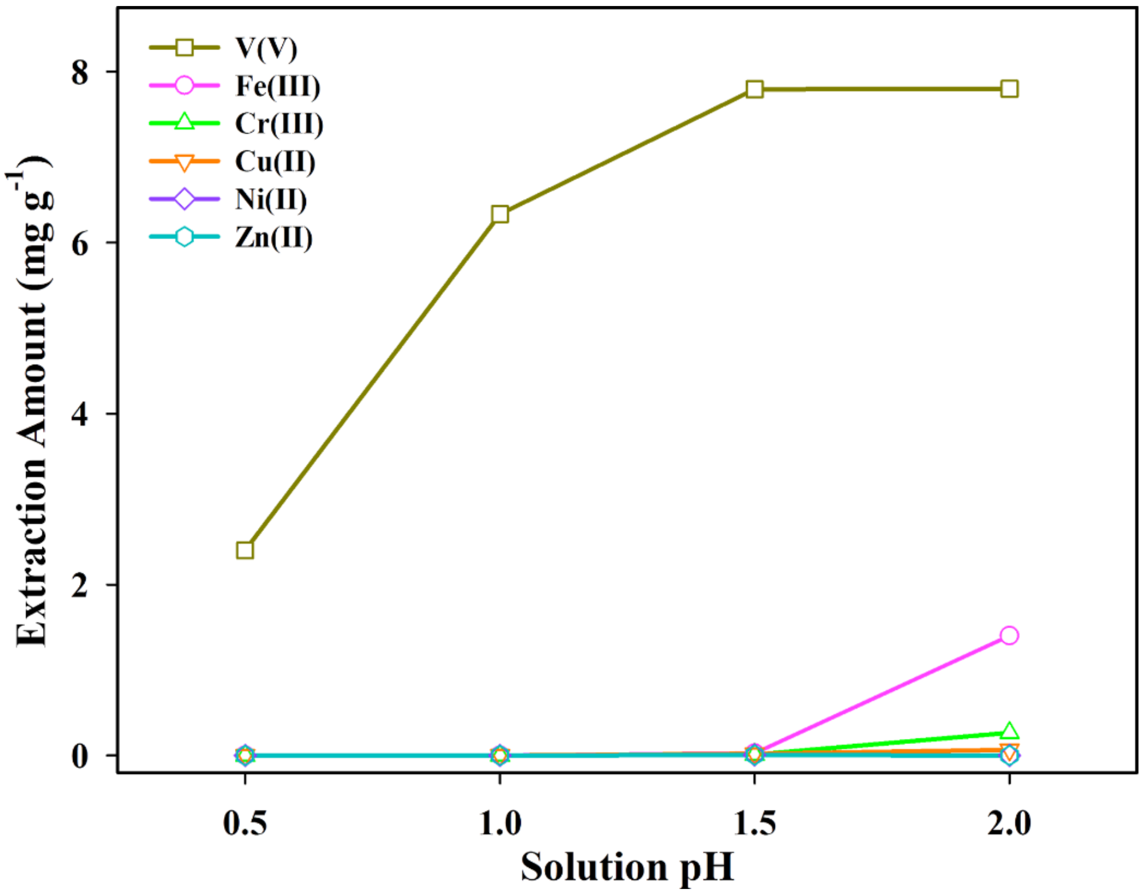

Supplementary Figure 20. Recovery of V(V) from mixed metal ion solutions under various pH. Source data are provided as a Source Data file.

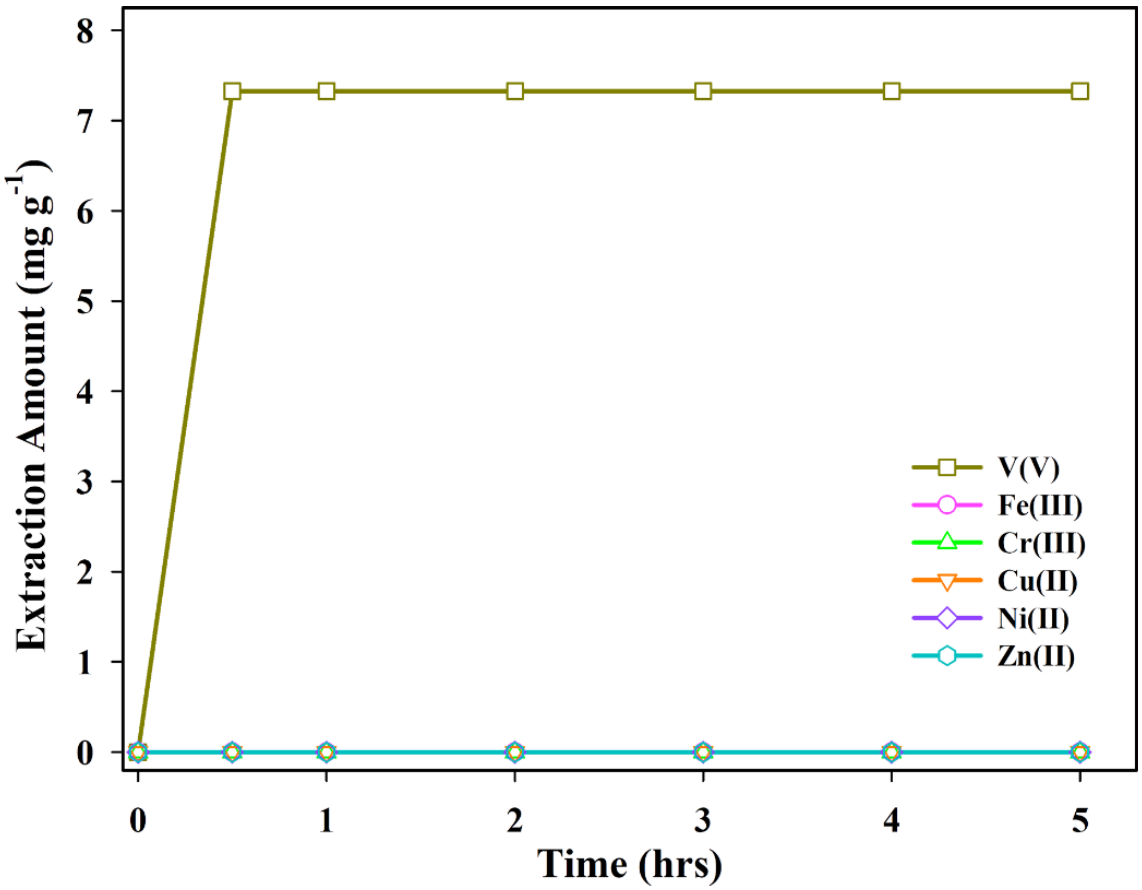

Supplementary Figure 21. The recovery kinetics of V(V) on H_2CID^{III} from a mixed metal ion solution (at pH = 1.5). Source data are provided as a Source Data file.

Supplementary Table 7. Comparison of the equiibrum uptake of V(V) and various other metal ions by H_2CID^{III}.

Ions	Capacity (mg/g)	V(V)/M^{n+} selectivity coefficient ($\beta_{V/competing\ metal}$)
V(V)	7.79	-
Fe(III)	0.02	2.9×10^5
Cr(III)	0.01	6.4×10^5
Cu(II)	0.02	4.3×10^6
Ni(II)	0.00	1.2×10^6
Zn(II)	0.00	1.3×10^6

Supplementary Table 8. V(V)/M^{n+} selectivity coefficient for H_2CID^{III} and literature V(V) capture materials

Adsorbent	Competing metal ion	V(V)/M^{n+} selectivity coefficient	Ref.
H_2CID^{III}	Fe(III)	2.9×10^5	This work
D2EHPA[1]	Fe(III)	4.7×10^2	29
TOMAC[2]+TBP[3]	Fe(III)	1.3×10^2	30
Cyanex 272[4]	Ni(II)	2.1×10^2	31
Tertiary amine N235	Fe(III)	1.0×10^3	32
Mextral 973H[5]	Fe(III)	7.2×10^2	33
N1923[6]	Cr(III)	5.4×10^2	34
LK-N21[7]	Cr(VI)	1.5×10^3	35
D2EHPA and EHEHPA[8]	Al(III)	1.4×10^2	36
[C8mim][PF6][9]	Cr(VI)	1.0×10^2	37
TOMAC[2] + 2-octanol	Fe(III)	2.4×10^3	30

[1] Di-2-ethylhexyl phosphoric acid

[2]tri-n-octylmethylammonium chloride

[3]Tributyl phosphate

[4]dialkyl phosphinic acid extractant

[5]2-hydroxy-5-nonylacetophenone oxime and 5-Nonylsalicylaldoxime

[6]$(C_{10}H_{21})_2CHNH_2$

[7]C_{16}-C_{20} primary amine

[8]di(2-ethylhexly)phosphoric acid (D2EHPA) and 2-ethylhexyl hydrogen -2-ethylhexylphosphonate (EHEHPA)

[9]1-octyl-3-methylimidazolium hexafluorophosphate

(a)

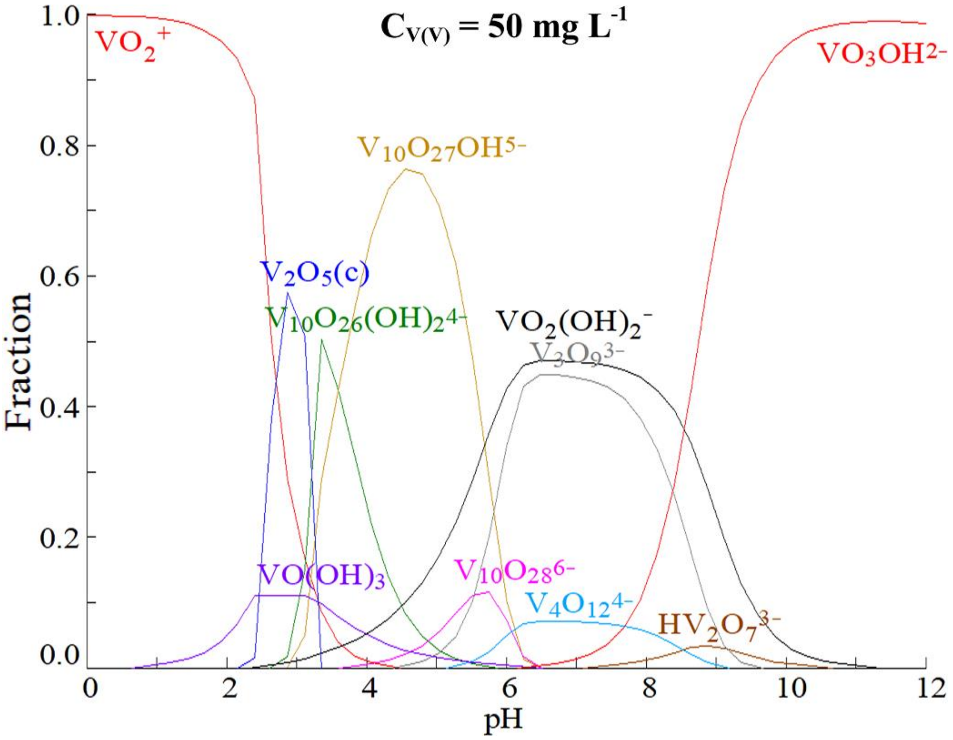

(b)

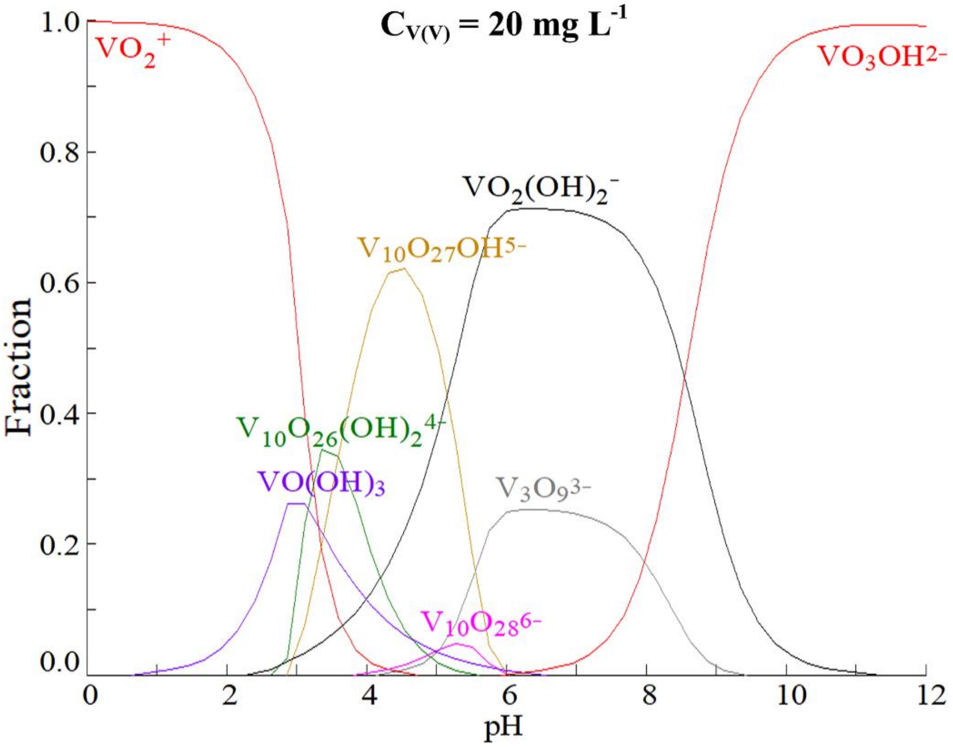

Supplementary Figure 22. The equilibrium diagrams for V(V) solutions. a 50 mg L^{-1} V(V) solution **b** 20 mg L^{-1} V(V) solution. The diagrams were obtained by the program of MEDUSA (Royal Institute of Technology, Sweden)

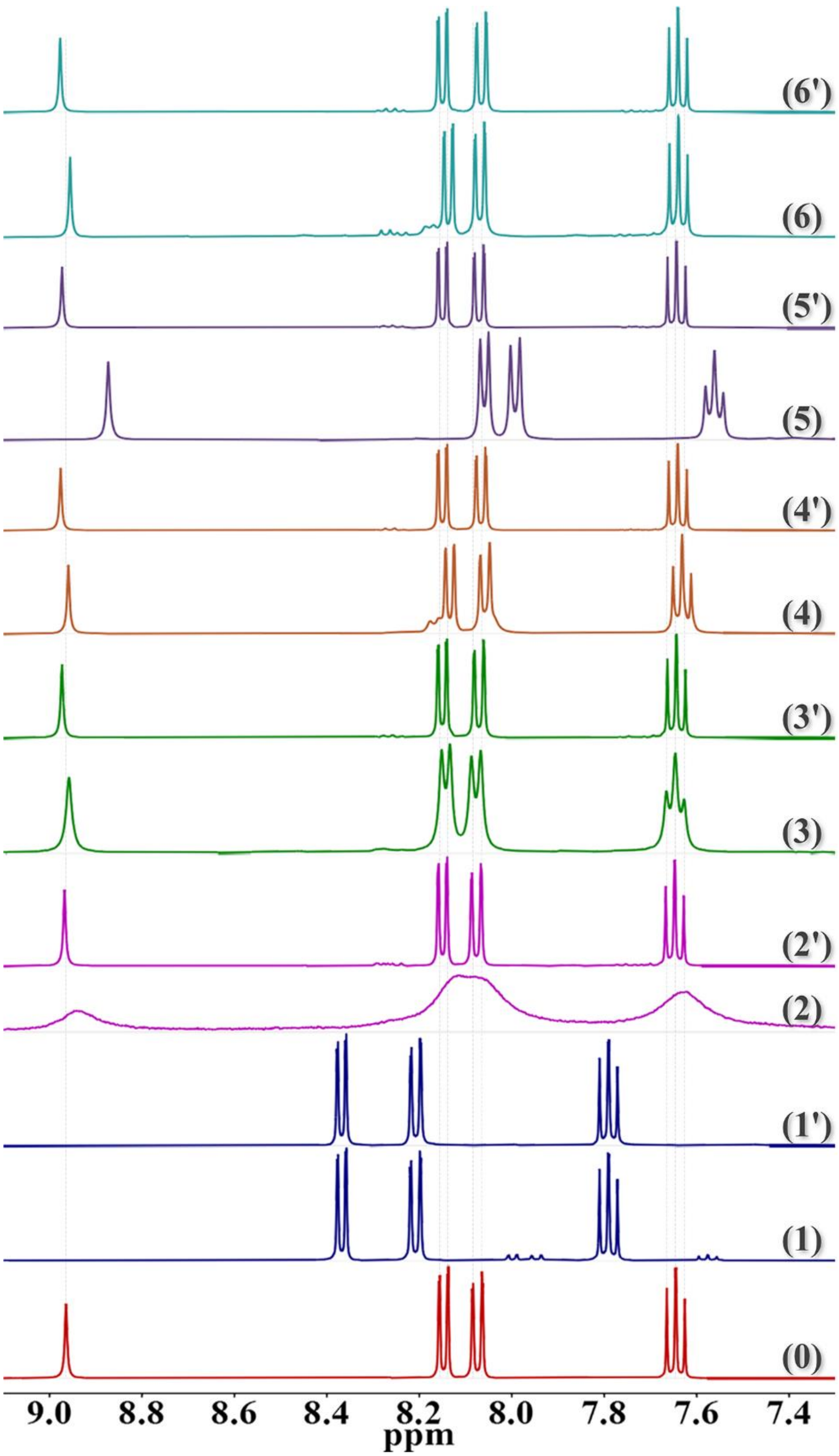

Supplementary Figure 23. ^{1}H NMR spectra of H_2CID^{III} (0), and V-CID^{III} complex (1, 1'), Fe-CID^{III} complex (2, 2'), Cr-CID^{III} complex (3, 3'), Cu-CID^{III} complex (4, 4'), Ni-CID^{III} complex (5, 5'), and Zn-CID^{III} complex (6, 6') before and after treatment with HNO_3 (pH 1.5) solution.

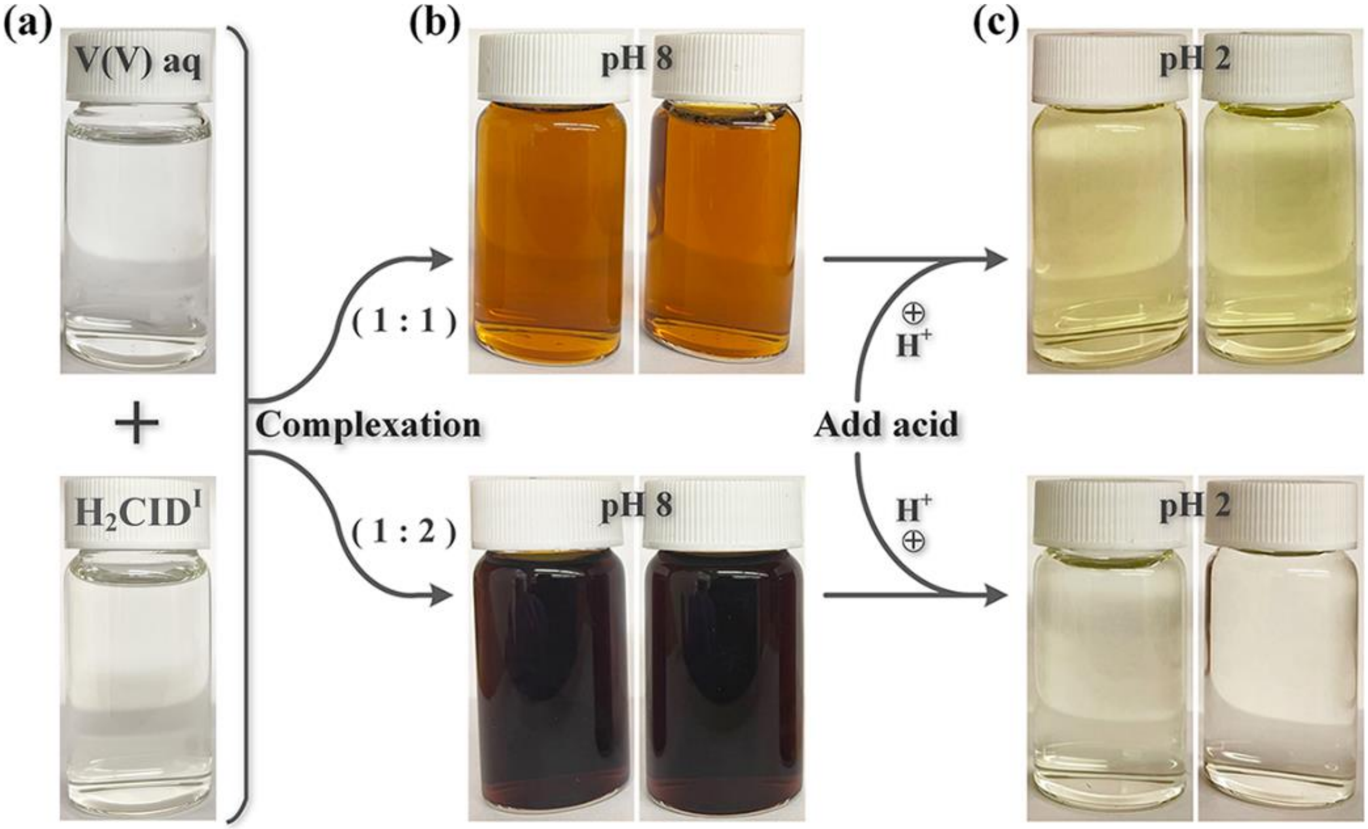

Supplementary Figure 24. Complexation and decomplexation of vanadium(V) with H_2CID^I. a V(V) and H_2CID^I aqueous solutions. **b** V(V)-complexes in solutions. **c** Solutions after pH adjustment from 8 to 2.

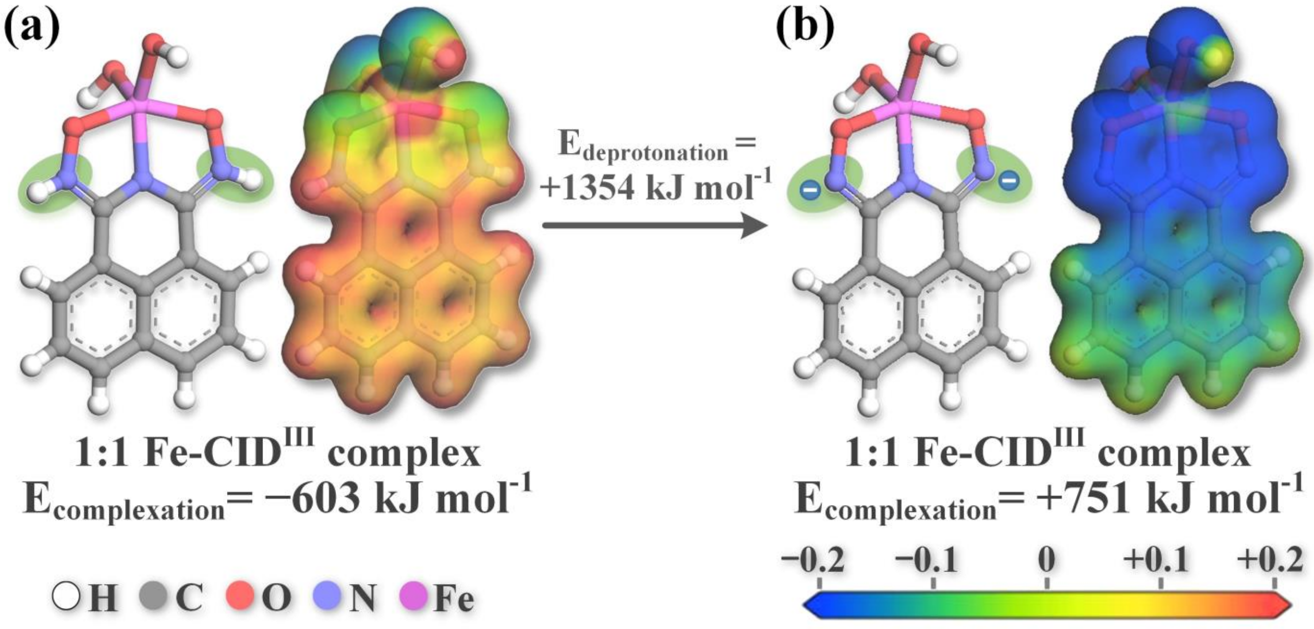

Supplementary Figure 25. $E_{complxation}$ and EPM. a protonated 1:1 Fe-CID^{III} complex. **b** deprotonated 1:1 Fe-CID^{III} complex.

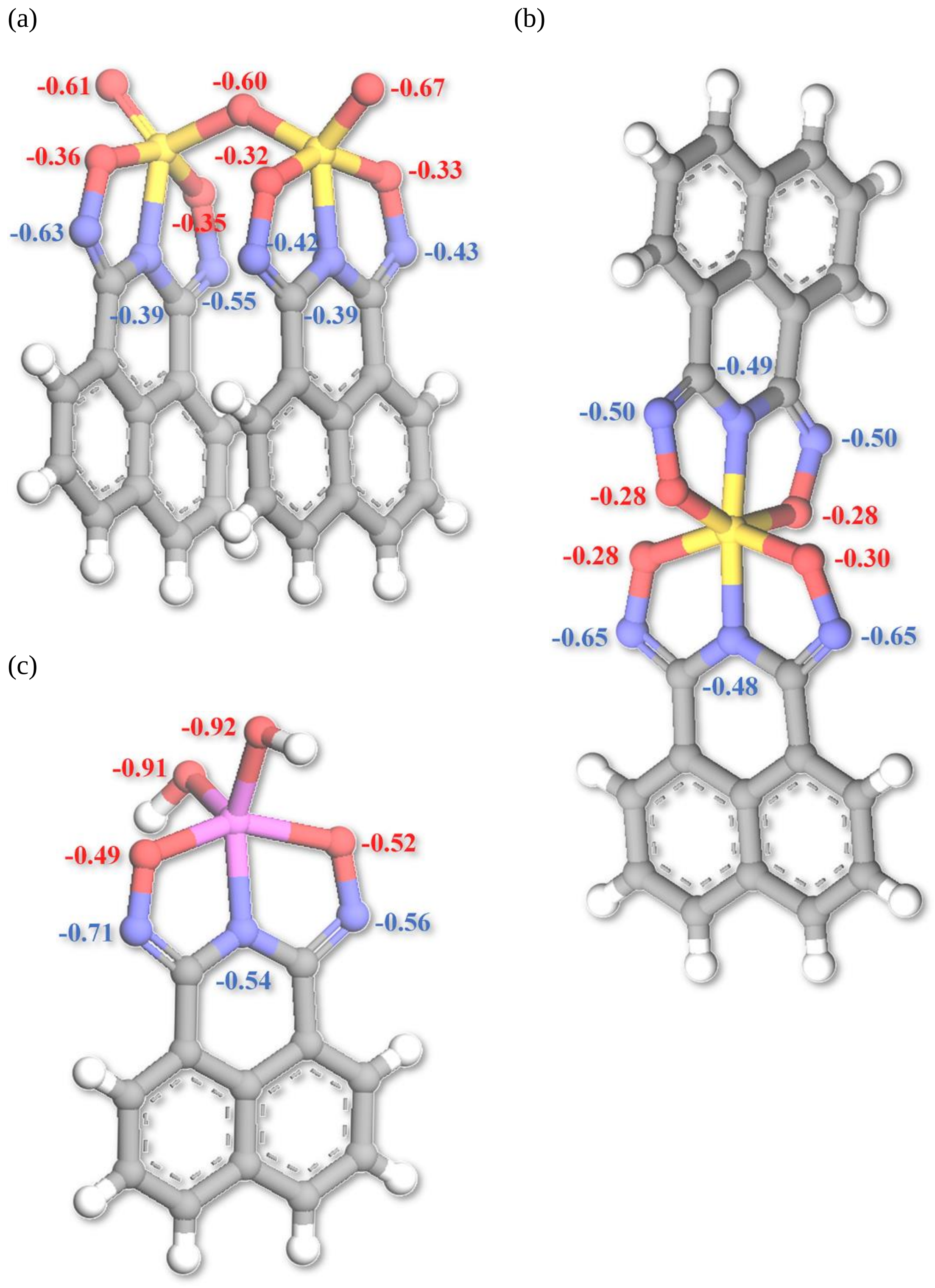

Supplementary Figure 26. Charges of oxygen (red) and nitrogen (blue) in V-complexes and Fe-complex. a-c. a 1:1 V-CID^{III} complex. **b** 1:2 V-CID^{III} complex. **c** 1:1 Fe-CID^{III} complex before neutralization.

Supplementary Table 9. Composition of the real waste source (solution pH 1.5)

Cations	Concentration (mg L^{-1})	Cations	Concentration (mg L^{-1})
Na	12465.0	Ba	3.18
Co	1332.5	Sr	2.16
Fe	763.6	Zn	1.84
Al	416.7	**V**	**1.57**
Ca	138.5	Cr	0.86
Ni	76.95	Pb	0.70
Mg	67.82	Th	0.19
K	57.20	Mo	0.17
Cu	20.87	Be	0.04
Mn	15.5	U	0.04

Supplementary Table 10. Comparison of the performance of H_2CID^{III} and several materials during the extraction of Vanadium from several sources

Material name	Waste source	pH	Capacity (mg/g)	Eff. (%)	time (hrs)	Competing ions	Remarks	Ref.
**Journal Articles**								
H_2CID^{III} (Chelating agent)	Oil sands tailings	1.5	205.6	95.5	2	Na, Co, Fe, Al, Ca, Ni, Mg, K, Cu, Mn, Ba, Sr, Zn, V, Cr, Pb, Th, Mo, Be, U	H_2CID^{III} exhibited high selectivity and affinity for V(V) at room temp., showcasing stability, reusability, and scalability.	*
DS418 (Ion exchange resin)	Vanadium shale	1.8	112.36	94.1	3	V, Al, Fe, K, Mg, Na, and P	The selectivity study solely targeted Fe competition	38
Bacterial cellulose (Agro-waste)	Simulated V solution	4	5.24	-	nr	-	Absence of stability, selectivity, and regeneration studies raises the potential for secondary waste generation.	39
Polymer inclusion membrane	Sim. spent hydrodesulphurization (HDS) catalysts solution	2.1	-	92.9	25	Mo, Co, Ni, Mn, and Al	The study's electrodialysis technology may be energy intensive. Also, the co-extracted Mo(IV), reduced V(V) affinity.	40

Lewatit Monoplus MP600 (Porous Anion-exchange resin)	Spent Catalysts	7	75.3	100	24	V, W	Extended extraction time and limited selectivity studies.	41
Oxalic acid and hexamethylenetetramine (HMT) (Complexing agent)	Carbon black	2.0	-	95.4	nr	V, Mo, Ni, Fe, and Ca	Extraction at 100°C for optimal efficiency.	42
Aminophosphonic acid chelating resin (Ion-exchange resin)	Black shale	1.8	-	90.1	nr	V, Fe, Al, K, Na, Mg, P	The extraction process duration was not revealed.	43
Microalgal strains	Simulated V(V) solution	4.3	-	59.9	24	-	Bacteria cultivated for seven days. The extraction process achieved low efficiency and long duration. Selectivity was not assessed.	44
Glycine (extractant - precipitation)	Simulated V(V) solution	1.8	-	95.7	1	-	High temp. (90°C) required for extraction	45
Bacillus mucilaginosus	Stone coal	5.0	-	44.4	18	-	Low extraction efficiency and long duration. Selectivity was not assessed.	46

Alamine 308	Spent hydro processing catalyst	1.5	-	99.8	0.08	V, Mo, Ni and Al	Co-extraction of Mo. Loss of effectiveness after the 4th cycle.	47
				**Patents**				
Calgon CPG	Crude or residual fuel oil	nr	-	81	~12	nr	Extraction performed at high temperature (80°C)	48
BMIM(l-butyl-3methylimidazolium) Acetate (Ionic liquid)	Organic residues from hydro-conversion processes	nr	-	58.1	24	nr	Extraction at 200°C; 58.1% V(V) extraction efficiency.	49
D201 resin and P507 extractant (an organophosphoric extractant)	Acid leached ores	0.2 and 1.8	-	51.9-100	1	Sc, Co, Ni, Mn, Cu, V, Mg	Co-extraction of impurities, necessitating subsequent purification steps. The study did not address the stability and reusability of the extractants.	50
Magnesium oxide, cobalt oxide, molybdenum oxide, activated carbon, zeolites or combinations thereof	Low-grade fuel	nr	-	99.7	nr	Ni, Fe and Al.	Co-extraction of Ni, Fe and Al. A high temperature (400-1000°F) was required for the extraction.	51

Electrolysis	Chromium ore, chromite	~1.2 and ~12	-	97.2	2	Cr	Co-extraction of Cr. High temperature (90°C) required	52
Quaternary ammonium polystyrene-divinyl benzene type (anion exchange resin)	Acid leach liquor	1.5	-	94	0.13	Fe	High temperature (65°C) required for the extraction process.	53

* - This work
nr – not reported

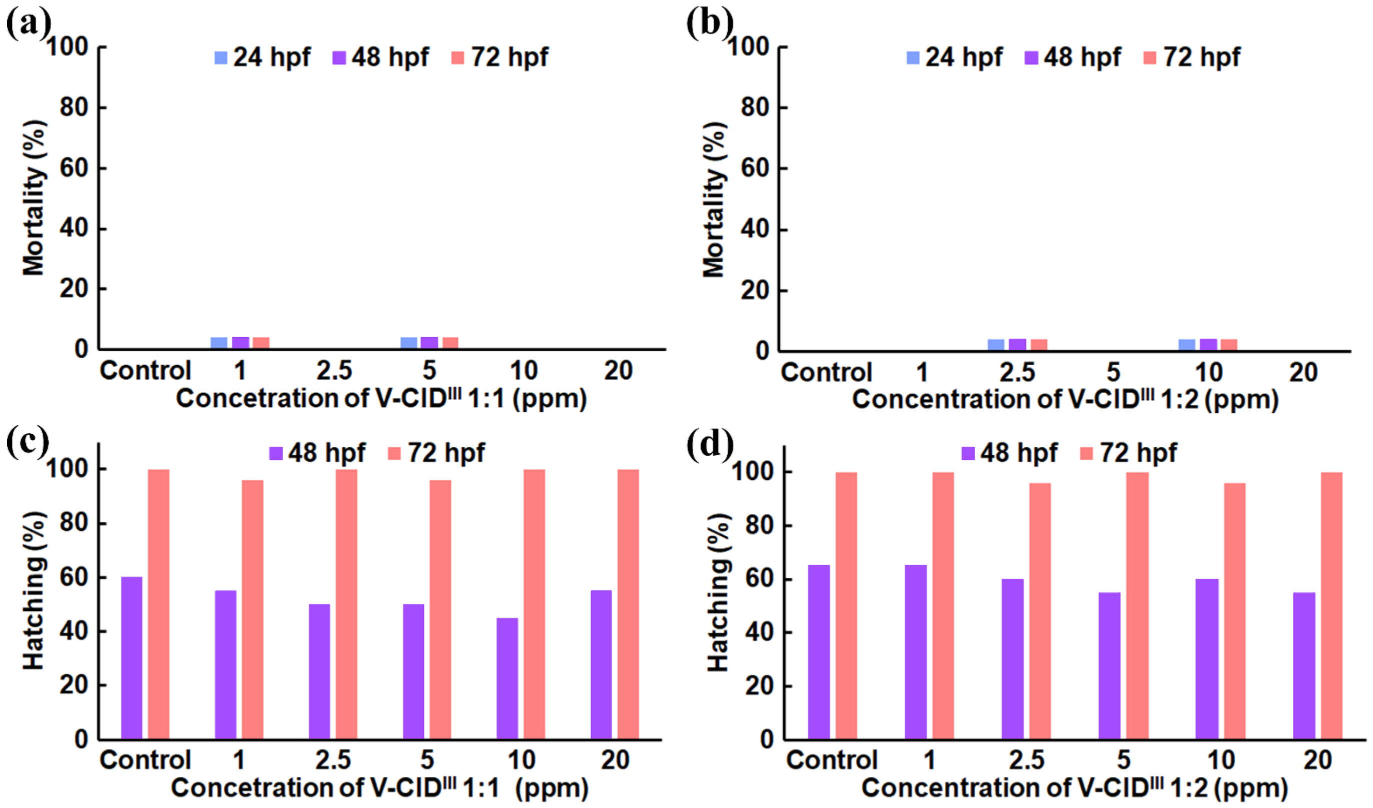

Supplementary Figure 27. Toxicity assessment. a and b Mortality rate (4%). **c and d** Hatching rate (~100%) of zebrafish embryos treated with **1** and **2**. Source data are provided as a Source Data file.

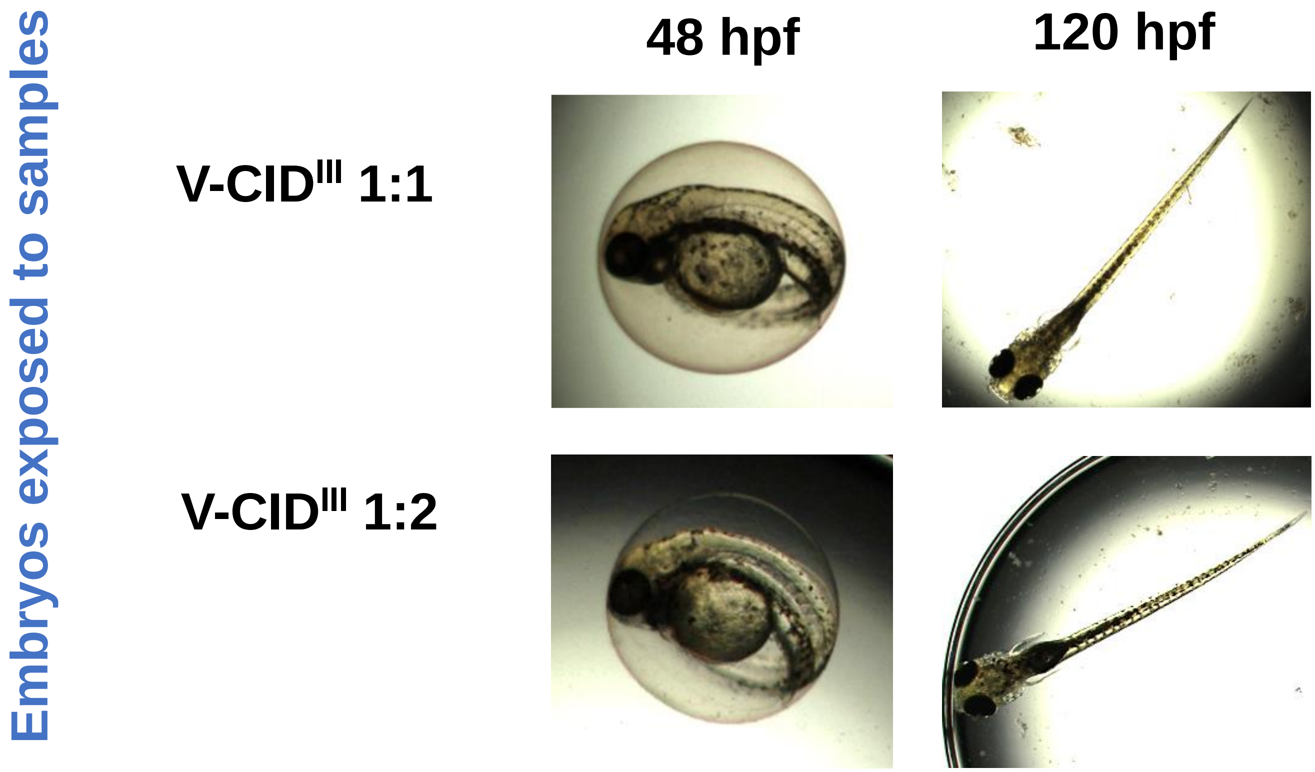

Supplementary Figure 28. Representative microscopic images of zebrafish embryos exposed to 1 and 2 (20 mg L^{-1}) obtained at 48 and 120 hpf.

Supplementary References

1. v. Braun, J. & Manz, G. Fluoranthen und seine Derivate. IV. *Justus Liebigs Ann. Chem.* **496**, 170–196 (1932).
2. Kang, S. O., Vukovic, S., Custelcean, R. & Hay, B. P. Cyclic Imide Dioximes: Formation and Hydrolytic Stability. *Ind. & Eng. Chem. Res.* **51**, 6619–6624 (2012).
3. Leggett, C. J. *et al.* Structural and spectroscopic studies of a rare non-oxido V(V) complex crystallized from aqueous solution. *Chem. Sci.* **7**, 2775–2786 (2016).
4. Lim, C. R., Lin, S. & Yun, Y. S. Highly efficient and acid-resistant metal-organic frameworks of MIL-101(Cr)-NH_2 for Pd(II) and Pt(IV) recovery from acidic solutions:*J. Hazard. Mater.* **387**, 121689 (2020).
5. Zhang, L., Liu, X., Xia, W. & Zhang, W. Preparation and characterization of chitosan-zirconium(IV) composite for adsorption of vanadium(V). *Int. J. Biol. Macromol.* **64**, 155–161 (2014).
6. Leiviskä, T., Khalid, M. K., Sarpola, A. & Tanskanen, J. Removal of vanadium from industrial wastewater using iron sorbents in batch and continuous flow pilot systems. *J. Environ. Manage.* **190**, 231–242 (2017).
7. Aregay, G. G. *et al.* Application of layered double hydroxide enriched with electron rich sulfide moieties ($S_2O_4^{2-}$) for efficient and selective removal of vanadium (V) from diverse aqueous medium. *Sci. Total Environ.* **792**, 148543 (2021).
8. Gogoi, H. *et al.* Vanadium removal by cationized sawdust produced through iodomethane quaternization of triethanolamine grafted raw material. *Chemosphere* **278**, 130445 (2021).
9. Liu, X. & Zhang, L. Insight into the adsorption mechanisms of vanadium(V) on a high-efficiency biosorbent (Ti-doped chitosan bead). *Int. J. Biol. Macromol.* **79**, 110–117 (2015).
10. Mazinai, A., Zare, K., Moradi, O. & Attar, H. Sulfonated calixarene modified Poly(methyl methacrylate) nanoparticles:A promising adsorbent for Removal of Vanadium Ions from aqueous media. *Chemosphere* **299**, 134459 (2022).
11. Zhu, X., Li, W. & Zhang, C. Extraction and removal of vanadium by adsorption with resin 201*7 from vanadium waste liquid. *Environ. Res.* **180**, 108865 (2020).
12. Ghanim, B. *et al.* Removal of vanadium from aqueous solution using a red mud modified saw dust biochar. *J. Water Process Eng.* **33**, 101076 (2020).
13. Salehi, S., Mandegarzad, S. & Anbia, M. Preparation and characterization of metal organic framework-derived nanoporous carbons for highly efficient removal of vanadium from aqueous solution. *J. Alloys Compd.* **812**, 152051 (2020).
14. Anirudhan, T. S., Jalajamony, S. & Divya, L. Efficiency of amine-modified poly(glycidyl methacrylate)-grafted cellulose in the removal and recovery of vanadium(V) from aqueous solutions.

Ind. Eng. Chem. Res. **48**, 2118–2124 (2009).

15. Ghanim, B. *et al.* Application of KOH modified seaweed hydrochar as a biosorbent of Vanadium from aqueous solution: Characterisations, mechanisms and regeneration capacity. *J. Environ. Chem. Eng.* **8**, 104176 (2020).
16. Li, M., Zhang, B., Zou, S., Liu, Q. & Yang, M. Highly selective adsorption of vanadium (V) by nano-hydrous zirconium oxide-modified anion exchange resin. *J. Hazard. Mater.* **384**, 121386 (2020).
17. Mahmoud, M. E., Abdelfattah, A. M. & Fekry, N. A. High performance of carbon quantum dots-decorated-polymeric nanocomposite for enhanced microwave adsorption of vanadium (V) from water. *Groundw. Sustain. Dev.* **14**, 100582 (2021).
18. Wołowicz, A. *et al.* Enhanced removal of vanadium(V) from acidic streams using binary oxide systems of TiO_2-ZrO_2 and TiO_2-ZnO type. *Sep. Purif. Technol.* **280**, 119916 (2022).
19. Solís–Rodríguez, R., Pérez–Garibay, R., Alonso–González, O., Mendieta–George, D. & Alvarado–Gómez, A. Vanadium removal by electrocoagulation with anodes of zinc. *J. Environ. Chem. Eng.* **10**, 108082 (2022).
20. Zulu, B., Oyewo, O. A., Sithole, B., Leswifi, T. Y. & Onyango, M. S. Functionalized Sawdust-Derived Cellulose Nanocrystalline Adsorbent for Efficient Removal of Vanadium From Aqueous Solution. *Front. Environ. Sci.* **8**, 1–11 (2020).
21. Thamilarasi, M. J. V., Anilkumar, P., Theivarasu, C. & Sureshkumar, M. V. Removal of vanadium from wastewater using surface-modified lignocellulosic material. *Environ. Sci. Pollut. Res.* **25**, 26182–26191 (2018).
22. Wan, X., Bao, S. & Zhang, Y. ZIF-8-derived porous carbon: application in capacitive deionization for vanadium (V) adsorption. *J. Appl. Electrochem.* **52**, 639–651 (2022).
23. He, W. yan, Liao, W., Yang, J. yan, Jeyakumar, P. & Anderson, C. Removal of vanadium from aquatic environment using phosphoric acid modified rice straw. *Bioremediat. J.* **24**, 80–89 (2020).
24. Zhao, Z., Li, X. & Zhao, Q. Recovery of V_2O_5 from Bayer liquor by ion exchange. *Rare Met.* **29**, 115–120 (2010).
25. Gao, Y. *et al.* BOF steel slag as a low-cost sorbent for vanadium (V) removal from soil washing effluent. *Sci. Rep.* **7**, 1–10 (2017).
26. Omidinasab, M. *et al.* Removal of vanadium and palladium ions by adsorption onto magnetic chitosan nanoparticles. *Environ. Sci. Pollut. Res.* **25**, 34262–34276 (2018).
27. Gomes, H. I., Jones, A., Rogerson, M., Burke, I. T. & Mayes, W. M. Vanadium removal and recovery from bauxite residue leachates by ion exchange. *Environ. Sci. Pollut. Res.* **23**, 23034–23042 (2016).
28. Chen, B., Bao, S., Zhang, Y. & Zheng, R. Ultrasound-assisted synthesis of N235- impregnated resins for vanadium (V) adsorption. *R. Soc. Open Sci.* **5**, 171746 (2018).

29. Tang, Y. *et al.* Solvent extraction of vanadium with D2EHPA from aqueous leachate of stone coal after low–temperature sulfation roasting. *Colloids Surfaces A Physicochem. Eng. Asp.* **650**, 129584 (2022).
30. Luo, D., Huang, J., Zhang, Y., Liu, H. & Hu, P. Efficient and environment-friendly vanadium (V) extraction from vanadium shale leachate using tri-n-octylmethylammonium chloride. *Sep. Purif. Technol.* **237**, 116482 (2020).
31. Noori, M., Rashchi, F., Babakhani, A. & Vahidi, E. Selective recovery and separation of nickel and vanadium in sulfate media using mixtures of D2EHPA and Cyanex 272. *Sep. Purif. Technol.* **136**, 265–273 (2014).
32. Ye, G., Hu, Y., Tong, X. & Lu, L. Extraction of vanadium from direct acid leaching solution of clay vanadium ore using solvent extraction with N235. *Hydrometallurgy* **177**, 27–33 (2018).
33. Zhang, Y. *et al.* Chelating extraction of vanadium(V) from low pH sulfuric acid solution by Mextral 973H. *Sep. Purif. Technol.* **190**, 123–135 (2018).
34. Wen, J. *et al.* Quantitative tuning of ionic metal species for ultra-selective metal solvent extraction toward high-purity vanadium products. *J. Hazard. Mater.* **425**, 127756 (2022).
35. Ning, P., Lin, X., Wang, X. & Cao, H. High-efficient extraction of vanadium and its application in the utilization of the chromium-bearing vanadium slag. *Chem. Eng. J.* **301**, 132–138 (2016).
36. Liu, H., Zhang, Y., Huang, J., Liu, T. & Wang, Y. Coordination extraction for separating vanadium and impurities from black shale HCl leachate at low pH using D2EHPA and EHEHPA mixture. *Sep. Purif. Technol.* **302**, 122088 (2022).
37. Hu, Q., Zhao, J., Wang, F., Huo, F. & Liu, H. Selective extraction of vanadium from chromium by pure [C8mim][PF_6]: An anion exchange process. *Sep. Purif. Technol.* **131**, 94–101 (2014).
38. Tan, L., Liu, T., Zhang, Y., Xue, N. & Hu, P. Enhance selective separation of vanadium and iron from acid-leaching solution with sulfamic acid modified resin. *Chem. Eng. J.* **457**, 141286 (2023).
39. Tseng, Y. S. *et al.* Removal of heavy metal vanadium from aqueous solution by nanocellulose produced from Komagataeibacter europaeus employing pineapple waste as carbon source. *Bioresour. Technol.* **369**, 128411 (2023).
40. Qin, Z. *et al.* Vanadium recovery by electrodialysis using polymer inclusion membranes. *J. Hazard. Mater.* **436**, 129315 (2022).
41. Jeon, J. H., Cueva Sola, A. B., Lee, J.-Y., Koduru, J. R. & Jyothi, R. K. Separation of vanadium and tungsten from synthetic and spent catalyst leach solutions using an ion-exchange resin. *RSC Adv.* **12**, 3635–3645 (2022).
42. Tan, H., Fan, B., Zheng, S. & Zhang, Y. Recovery and Separation of Vanadium, Nickel, and Molybdenum from the Industrial Waste of a Petroleum Refinery by a Complexation Method. *ACS Sustain. Chem. & Eng.* **11**, 4894–4902 (2023).

43. Wang, L., Xue, N., Zhang, Y. & Zheng, Q. Selective separation and recovery of vanadium from acid leaching solution of polymetallic black shale as function of aminophosphonic acid. *J. Chem. Technol. & Biotechnol.* **97**, 1978–1986 (2022).
44. Kim, H. S. *et al.* Microalgae as an Effective Recovery Agent for Vanadium in Aquatic Environment. *Energies* **15**, 4467 (2022).
45. Peng, H. *et al.* Vanadium recovery by glycine precipitation. *Environ. Chem. Lett.* **20**, 1569–1575 (2022).
46. Dong, Y., Chong, S. & Lin, H. Bioleaching and biosorption behavior of vanadium-bearing stone coal by Bacillus mucilaginosus. *Int. J. Miner. Metall. Mater.* **30**, 283–292 (2022).
47. Sahu, K. K., Agrawal, A. & Mishra, D. Hazardous waste to materials: Recovery of molybdenum and vanadium from acidic leach liquor of spent hydroprocessing catalyst using alamine 308. *J. Environ. Manage.* **125**, 68–73 (2013).
48. Silva J, P. C. and L. G. Adsorption of vanadium compounds from fuel oil and adsorbents thereof. US20080169221 (2008).
49. Guidetti, S. & De Angelis, A. Process for the selective recovery of transition metals from organic residues. WO2020121220 (2020).
50. Gauthier, P. & Di Cesare, E. Recovery of scandium and vanadium values from feedstocks using ultrasound-assisted extraction. WO2018232528 (2018).
51. Kulkarni, P. Methods and systems for removing vanadium from low-grade fuels. US20090057202 (2009).
52. Weber, R., Block, H. & Batz, M. Process for the utilization of vanadium bound in chromium ore as vanadium (V) oxide by electrolysis. GB2387840B (2003).
53. McLean, D. Multistage adsorption of pentavalent vanadium values on anion exchange resins. US2937072A (1960).
